# MiR-422a regulates cellular metabolism and malignancy by targeting pyruvate dehydrogenase kinase 2 in gastric cancer

**DOI:** 10.1038/s41419-018-0564-3

**Published:** 2018-05-02

**Authors:** Zhongyuan He, Zheng Li, Xuan Zhang, Kai Yin, Weizhi Wang, Zhipeng Xu, Bowen Li, Lei Zhang, Jianghao Xu, Guangli Sun, Lu Wang, Qing Li, Xiaoxu Huang, Lu Zhang, Diancai Zhang, Hao Xu, Zekuan Xu

**Affiliations:** 1 Department of General Surgery, The First Affiliated Hospital of Nanjing Medical University, Nanjing, Jiangsu Province 210029 China; 2 Department of General Surgery, Affiliated Hospital of Jiangsu University, Zhenjiang, Jiangsu Province 212004 China; 3 Collaborative Innovation Center For Cancer Personalized Medicine, Nanjing Medical University, Nanjing, Jiangsu Province 210029 China

## Abstract

Increasing evidence indicates that dysregulation of microRNAs (miRNAs) plays a crucial role in human malignancies. Here, we showed that microRNA-422a (miR-422a) expression was dramatically downregulated in gastric cancer (GC) samples and cell lines compared with normal controls, and that its expression level was inversely related to tumor size and depth of infiltration. Functional studies revealed that the overexpression of miR-422a in GC tumor cells suppressed cell proliferation and migration, and drove a metabolic shift from aerobic glycolysis to oxidative phosphorylation. Mechanistic analysis suggested that miR-422a repressed pyruvate dehydrogenase kinase 2 (PDK2) to restore activity of the pyruvate dehydrogenase (PDH), the gatekeeping enzyme that catalyzes the decarboxylation of pyruvate to produce acetyl-CoA. Importantly, we further demonstrated that the mir-422a–PDK2 axis also influenced another metabolic pathway, de novo lipogenesis in cancer cells, and that it subsequently affected reactive oxygen species (ROS) and RB phosphorylation levels, ultimately resulting in cell cycle arrest in G1 phase. Our findings show that the miR-422a–PDK2 axis is an important mediator in metabolic reprogramming and a promising therapeutic target for antitumor treatment.

## Introduction

Gastric cancer (GC), the fifth most frequently diagnosed malignancy and the third-ranked cause of cancer-related deaths worldwide, displays considerable regional disparity^1^. Despite the gradually declining incidence of GC, the 5-year survival rate of patients with GC is only 20–30%^2^. The tumorigenesis and progression of GC are affected by multiple events through which cells undergo a series of genetic and epigenetic transformations of pivotal growth regulatory genes that confer proliferative and survival advantages on the cells^3,4^. Hence, a more comprehensive understanding of the molecular mechanisms underlying GC disease pathways would contribute to the development of novel preventive, therapeutic and diagnostic methods for cancer.

MicroRNAs (miRNAs) are small noncoding RNAs that post-transcriptionally modulate gene expression via binding to the 3′-untranslated region (UTR) of target mRNAs, resulting in their degradation or translational suppression. Accumulating evidence indicates that miRNAs are involved in a wide range of physiological and pathological processes, including tumor initiation and progression^5,6^. Therefore, miRNAs have been proposed as potential prognostic biomarkers and therapeutic targets for GC^7^. Despite its having been characterized as a tumor-suppressor gene for lung cancer and colorectal cancer, the biological functions of microRNA-422a (miR-422a) and its molecular mechanisms in GC remain unknown.

Cancer cells undergo metabolic reprogramming that enables them to primarily utilize glucose for energy production, a phenomenon known as the Warburg effect^8^. In addition to producing ATP, enhanced glycolysis generates glycolytic intermediates that are required by fast-growing tumors^9–11^. Although it is well accepted that the Warburg effect occurs in GC, the mechanism driving aerobic glycolysis in this cancer remains largely unknown. Therefore, searching for the deep mechanism is urged for therapeutic aims. Previous studies demonstrated that miRNAs play regulatory roles in the metabolism of cancer cells^12–14^. In regard to GC, however, little is known of the effects of miRNAs on glucose metabolism. In addition to aerobic glycolysis, cancer cells also display abnormalities in other metabolic processes, including oxidative phosphorylation, glutaminolysis and lipogenesis^15–17^. These metabolic pathways also provide cancer cells with energy in the form of ATP and with various metabolites, including nucleotides, amino acids and lipids, as the building blocks for accelerated cell division. For example, lipids are the most important components of membranes and participate in many important cancer-associated signaling pathways as second messengers or through the modification of key enzymes^18,19^.

Reactive oxygen species (ROS) are formed as a natural byproduct of the normal metabolism of oxygen and have important roles in cell signaling and homeostasis^20–22^. Excessive ROS production results in apoptosis and cell cycle arrest in cancer^23–25^.

In this study, we showed that miR-422a acts as an effective suppressor of the Warburg effect by targeting pyruvate dehydrogenase kinase 2 (PDK2). In addition to repressing aerobic glycolysis of GC tumor cells, the miR-422a–PDK2 axis promoted lipogenesis and elevated the production of ROS, leading to rapid hypophosphorylation of retinoblastoma protein (RB) and cell cycle arrest.

## Results

### MiR-422a expression in GC samples and cell lines is downregulated via epigenetic mechanisms

**Fig. 1:**
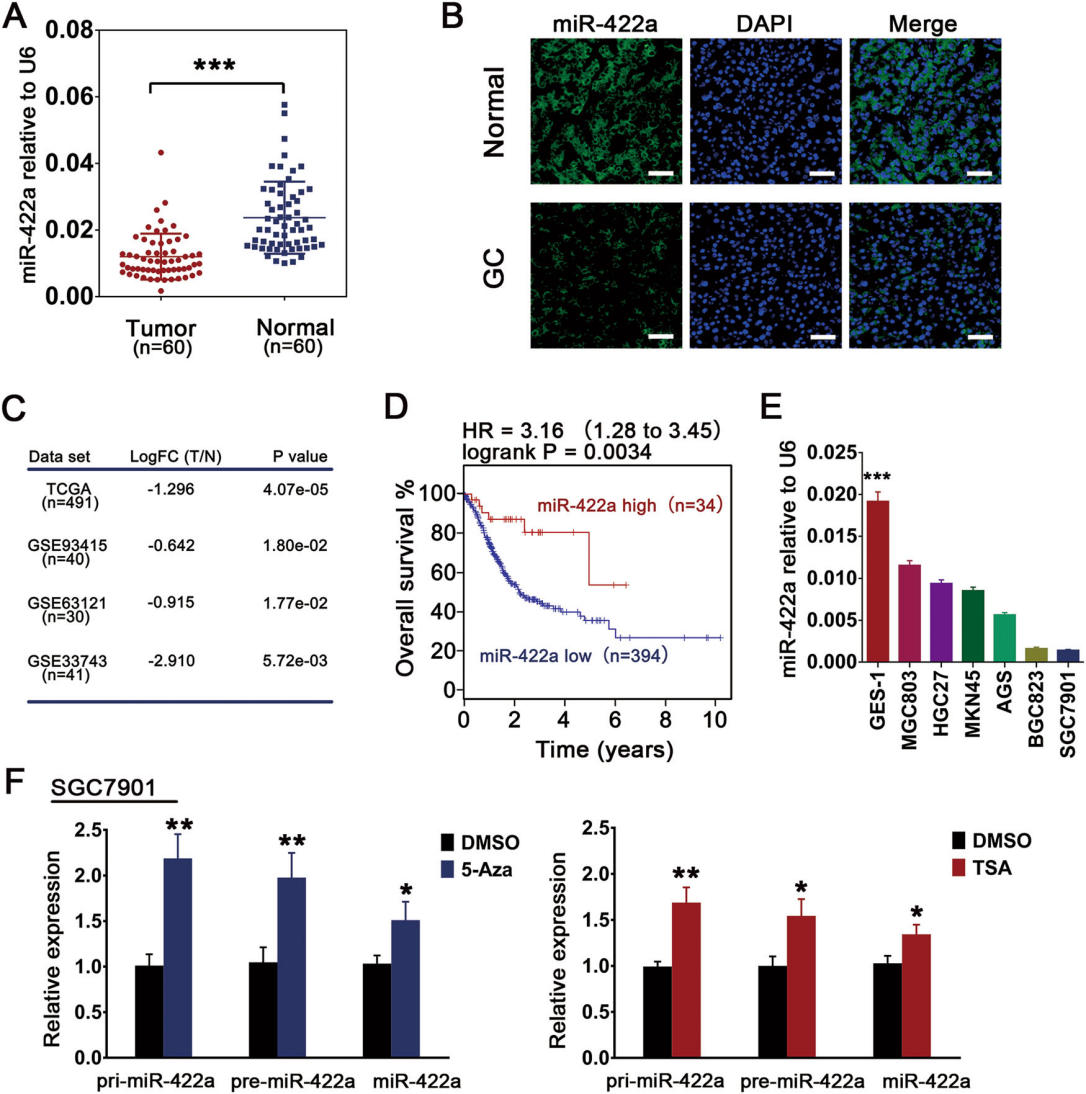
miR-422a expression is frequently downregulated in gastric cancer and regulated by epigenetic events. **a** miR-422a expression level in GC tissues and matched noncancerous gastric mucosa tissues analyzed by qRT-PCR. **b** Representative FISH images of miR-422a expression. Scale bars, 50 μm. **c** Comparison of miR-422a expression in GC tissues and normal gastric mucosal tissues in the TCGA and GEO databases. **d** Overall survival analysis of patients with high or low miR-422a expression in the TCGA database. **e** qRT-PCR analysis of miR-422a expression in all six GC cell lines and in the normal human gastric epithelial cell line GES-1. One-way ANOVA and Dunnett’s multiple comparison test. **f** After incubation with 2.5 μM 5-aza-2′-deoxycytidine (5-Aza) for 72 h or with 100 ng/ml trichostatin A (TSA) for 12 h, relative expression levels of pri-miR-422a, pre-miR-422a, and mature miR-422a in SGC7901 cells were determined by RT-PCR. GAPDH or U6 were used for normalization of pri-/ pre-miR-422a and mature miR-422a, respectively. The error bars represent the mean (*n* = 3) ± S.D. *P < 0.05, **P < 0.01, ***P < 0.001 versus corresponding NC

We first measured miR-422a expression using quantitative reverse transcriptase-PCR (qRT-PCR) in 60 paired tumor tissues and in corresponding adjacent tissues from GC patients. The results revealed that miR-422a expression in the normal tissues was 1.95-fold higher than that in the matched GC tissues (*P* < 0.0001) (Fig. 1a). And we obtained consistent results from fluorescence in situ hybridization (FISH) analysis (Fig. 1b). Then, we analyzed miR-422a expression in four previously published microarray data sets obtained from GC samples deposited in the TCGA portal and NCBI GEO (GSE93415, GSE63121, GSE33743). In these data sets, miR-422a was significantly downregulated in tumor samples compared with normal tissues (Fig. 1c). Additionally, the data from TCGA indicated that patients with high miR-422a expression survived longer than those with low expression (Fig. 1d). Finally, miR-422a was weakly expressed in the GC cell lines compared with its expression in normal gastric mucosa cell line GES-1 (Fig. 1e).

The clinicopathologic features of the GC patients are summarized in Table 1. Decreased miR-422a expression was inversely correlated with tumor size and depth of infiltration (*P* = 0.020 and 0.037, respectively).

**Table 1:** Correlation between relative miR-422a expression and the clinicopathological characteristics of patients with gastric cancer

Characteristics		Number	No. of patients		*P*-value
			miR-422a^high^	miR-422a^low^	
Age (y)	≥60	45	20	25	0.136
	<60	15	10	5	
Gender	Male	44	21	23	0.559
	Female	16	9	7	
Tumor size (cm)	≥3.5	29	10	19	0.020
	<3.5	31	20	11	
Histological grade	Well moderately	21	10	11	0.787
	Poorly signet	39	20	19	
Clinical stage	I–II	35	18	17	0.793
	III–IV	25	12	13	
T classification	T1–T2	26	17	9	0.037
	T3–T4	34	13	21	
N classification	N0	26	14	12	0.969
	N1–N3	34	16	14	

Accumulating evidence supports the idea that epigenetic mechanisms such as DNA methylation control the expression of miRNA genes in various cancers^26–28^. Interestingly, pri-/pre- and mature miR-422a expression in the SGC7901 and BGC823 cell lines increased significantly after treatment of the cells with 5-aza-2′-deoxycytidine (5-Aza), an inhibitor of DNA methyltransferase, or trichostatin A (TSA), an inhibitor of the class I and II HDAC families (Fig. 1f and Supplementary Figure 1A). The genomic locus of miR-422a was evaluated using online software (www.urogene.org); there were two regions with a high frequency of CpG sites (Supplementary Figure 1B). Bisulfate genomic sequencing (BSP) assay revealed that the methylation levels in those two regions of the miR-422a promoter were upregulated in the SGC7901 and BGC823 cell lines (Supplementary Figure 1C).

These findings suggested that miR-422a may act as a tumor suppressor in GC and verify the involvement of epigenetic regulation in miR-422a silencing.

### MiR-422a suppresses tumor proliferation and metastasis both in vitro and in vivo

**Fig. 2:**
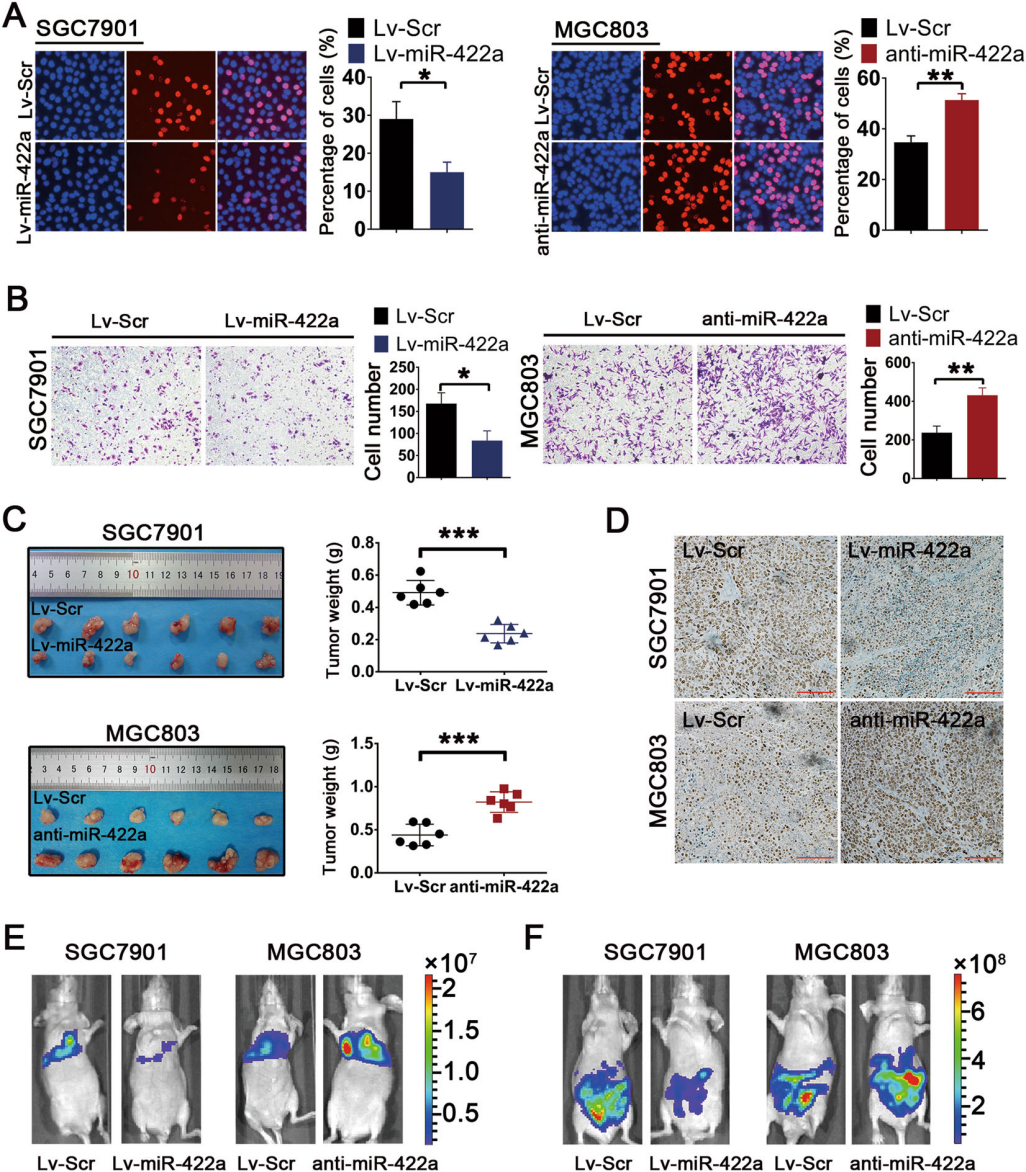
In vitro and in vivo functional analysis of miR-422a. **a** DNA synthesis in GC cells was measured using the Edu incorporation assay. Red fluorescence represents Edu-positive cells; blue fluorescence from Hoechst stain represents the total cells. **b** Transwell assays were performed to evaluate the effect of miR-422a on cell migration. Cells were counted under a microscope in five randomly selected fields. **c** The in vivo effect of miR-422a was evaluated in xenograft mouse models bearing tumors originating from SGC7901 and MGC803 cells; *n* = 6 per group. **d** Representative Ki67 staining of primary tumor tissues. Scale bar: 50 μm. **e** Bioluminescent images of mice administered with SGC7901 and MGC803 cells via the lateral tail veins. Images were taken after 28 days. *n* = 6 per group. **f** Bioluminescent images of the metastatic tumors in mice 28 days after intraperitoneal injection with GC cells. *n* = 6 per group. The error bars represent the mean (*n* = 3) ± S.D. *P < 0.05, **P < 0.01, ***P < 0.001 versus corresponding NC

We restored miR-422a expression in the SGC7901 and BGC823 cell lines, and suppressed its expression in the MGC803 and HGC27, by lentivirus infection. Using qRT-PCR analysis, the efficient impact of lentivirus infection was confirmed (Supplementary Figure 2A). The Edu and colony formation assays revealed that overexpression of miR-422a markedly repressed GC cell proliferation, whereas suppression of miR-422a significantly promoted cell growth (Fig. 2a and Supplementary Figure 2D). Furthermore, we evaluated the effects of miR-422a on tumor cell motility in vitro using Transwell chambers. Ectopic expression of miR-422a significantly decreased cellular migration. In contrast, cell migration was markedly enhanced after inhibition of miR-422a (Fig. 2b and Supplementary Figure 2E). These results show that miR-422a affects cell proliferation and migration in vitro.

Next, we examined the in vivo roles of miR-422a in GC tumor cells. A nude mouse xenograft model was constructed by subcutaneous injection of SGC7901 cells overexpressing miR-422a or of MGC803 cells with miR-422a deletion. Tumor volume and Ki67 expression were significantly decreased after miR-422a overexpression in SGC7901 cells, whereas opposing results were obtained in MGC803 cells with miR-422a deletion (Figs. 2c, d). To further quantify the impact of miR-422a on metastatic and dissemination potential in vivo, GC cells were injected into the lateral tail veins of nude mice or intraperitoneally transplanted into nude mice. Ectopic expression of miR-422a significantly inhibited metastasis potential and peritoneal dissemination in vivo, whereas miR-422a knockdown in MGC803 cells significantly promoted both (Figs. 2e, f). These in vivo data strongly indicate the role of miR-422a as a suppressor of tumor growth.

### MiR-422a inhibits the Warburg effect

**Fig. 3:**
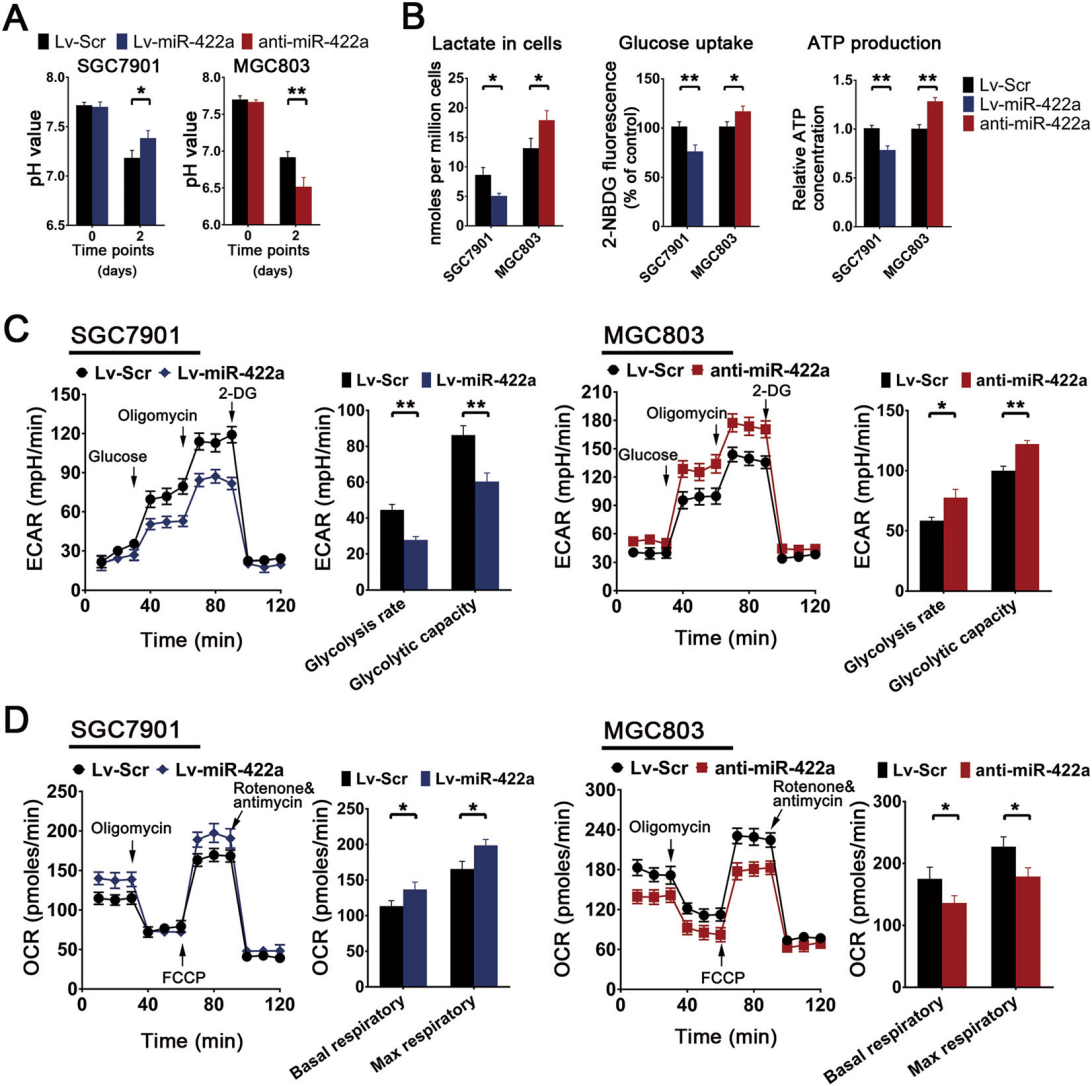
Analysis of miR-422a effects on glucose metabolism. **a** The pH of the culture medium was measured at the indicated time points (day 0 and day 2). **b** Lactate, ATP production and glucose uptake analysis of GC cell lines were determined via flow cytometry and the colorimetric method, respectively. **c** Analysis of extracellular acidification rate of miR-422a-overexpressing SGC7901 cells (left) and MGC803 cells with miR-422a knockdown (right). The extracellular acidification rate after glucose treatment indicates the rate of glycolysis. The extracellular acidification rate after oligomycin treatment indicates the glycolytic capacity. **d** Analysis of oxygen consumption rate of miR-422a-overexpressing SGC7901 cells (left) and MGC803 cells with miR-422a knockdown (right). The oxygen consumption rate before oligomycin treatment indicates the basal respiratory rate. The oxygen consumption rate after FCCP treatment indicates the maximum respiratory rate. The error bars represent the mean (*n* = 3) ± S.D. *P < 0.05, **P < 0.01 versus corresponding NC

In the course of culturing the GC cell lines, we found that in MGC803 cell and HGC27 cultures in which miR-422a expression had been repressed, the color of the culture medium changed from pink to orange more rapidly and the pH of the medium decreased from 7.7 to 6.5 at day 2; in contrast, overexpression of miR-422a in SGC7901 and BGC823 cells markedly inhibited the decrease in the pH of the medium that normally occurs with cell growth (Fig. 3a, Supplementary Figure 2B and 3A). To exclude the possibility that the variations in pH resulted from a difference in cell numbers, we performed CCK-8 assays and found that optical density (OD) values displayed no changes at day 2 after miR-422a overexpression or knockdown (Supplementary Figure 2C). Based on this observation, we hypothesized that miR-422a might regulate glycolysis and subsequently lactate production in GC cells. As expected, forced miR-422a expression decreased lactate, ATP production and glucose uptake in SGC7901 and BGC823 cells, whereas the opposite results were obtained in MGC803 and HGC27 cells with miR-422a knockdown (Fig. 3b and Supplementary Figure 3B). To verify the impact of miR-422a on glycolysis and oxidative phosphorylation, extracellular acidification rates (ECARs) and oxygen consumption rates (OCR) were measured. Overexpression of miR-422a markedly decreased the glycolytic capacity and the glycolysis rate of miR-422a-overexpressing SGC7901 and BGC823 cells, whereas miR-422a knockdown in MGC803 and HGC27 cells significantly enhanced both (Fig. 3c and Supplementary Figure 3C). Moreover, the mitochondrial function of oxidative phosphorylation was also affected by miR-422a levels, as reflected by the alternation in oxygen consumption and respiratory capacity. MiR-422a overexpression resulted in a markedly enhanced basal OCR and significantly elevated maximum OCR relative to controls. On the other hand, miR-422a knockdown led to lower OCR at both basal and maximal levels (Fig. 3d and Supplementary Figure 3D). These data collectively suggest that miR-422a made contributes greatly to the occurrence of a cellular metabolic shift between glycolysis and oxidative phosphorylation.

### Pyruvate dehydrogenase (PDK2) is a direct functional target of miR-422a in GC

**Fig. 4:**
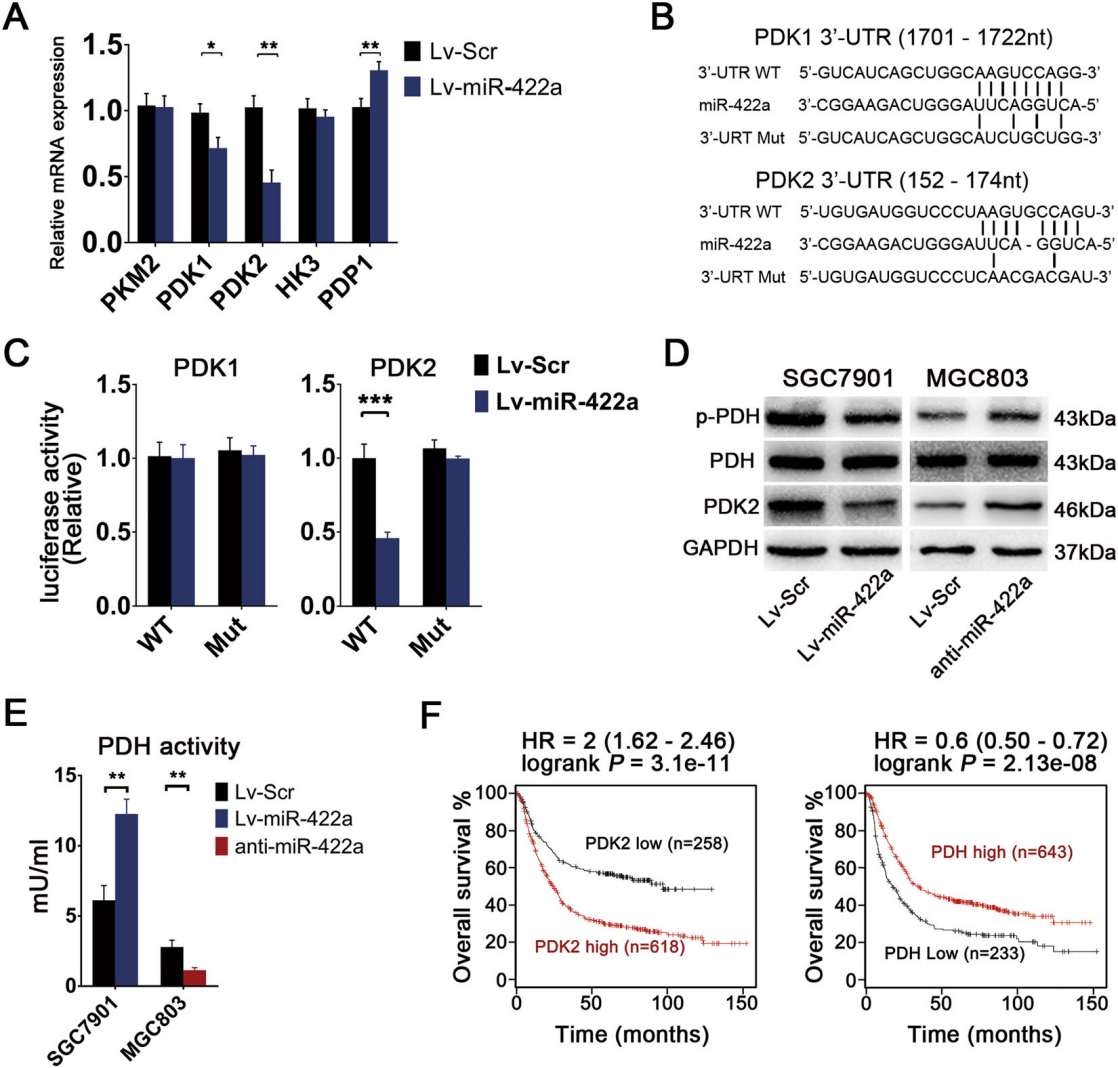
Identification of PDK2 as an miR-422a target in GC. **a** qRT-PCR analysis of five candidate genes after miR-422a overexpression in SGC7901 cells. **b** miR-422a targeting sites in the 3′-UTR of targets genes and corresponding mutations. **c** A luciferase reporter assay was used to evaluate interactions between miR-422a and the targeting sites of candidate genes in SGC7901 cells. **d** Levels of PDK2, total PDH and phosphorylated PDH-E1α protein in cancer cells with miR-422a overexpression or knockdown. **e** After miR-422a overexpression in SGC7901 and miR-422a knockdown in MGC803 cells, the cells were harvested for measurement of cellular PDH activity. **f** Overall survival analysis of the patients based on PDK2 and PDH expression in the KM-plotter database. The error bars represent the mean (*n* = 3) ± S.D. *P < 0.05, **P < 0.01, ***P < 0.001 versus corresponding NC

To determine the underlying molecular mechanism through which miR-422a regulates the cellular metabolism in GC cells, several algorithms were applied to investigate putative targets of miR-422a. Of note, PKM2, PDK1, PDK2, HK3, PDP1 and PDHX are directly involved in glucose metabolism (Supplementary Figure 4A). To confirm the actual targets of miR-422a among these candidate genes, qRT-PCR analysis was performed in the miR-422a-overexpressing SGC7901 cell line and in control cells. The expression of PDK1 and PDK2 was significantly downregulated in the miR-422a-overexpressing cell line (Fig. 4a). To test whether the predicted miR-422a-binding sites in the PDK1 and PDK2 3′-UTR regions are subjected to regulation by miR-422a, we cloned their wild-type (WT) and mutated 3′-UTR regions into pGL3 downstream from a luciferase reporter gene (Fig. 4b) and transfected these vectors into miR-422a-overexpressing SGC7901 cells and control cells. The luciferase activity of the PDK2 reporter was significantly suppressed by ectopic expression of miR-422a, whereas the luciferase activity of the PDK1 reporter displayed no change (Fig. 4c). Meanwhile, the correlation between PDK2 expression and expression of miR-422a was determined in the same tumor tissues that were used to obtained the data shown in Fig. 1a (Supplementary Figure 4D). As expected, overexpression of miR-422a markedly decreased the protein level of endogenous PDK2, leading to a reduction in the phosphorylation of PDH-E1α and to a marked increment in PDH activity. An opposite result was obtained after miR-422a deletion (Figs. 4d, e, Supplementary Figure 4B and 4C). These results reveal that PDK2 is a direct functional target of miR-422a in GC tumorigenesis. Moreover, in the KM-plotter database^29^, lower PDK2 expression and higher PDH expression were significantly associated with extended patient survival (Fig. 4f).

### PDK2’s functions in tumor proliferation and cellular metabolism are opposite to those of miR-422a in GC

**Fig. 5:**
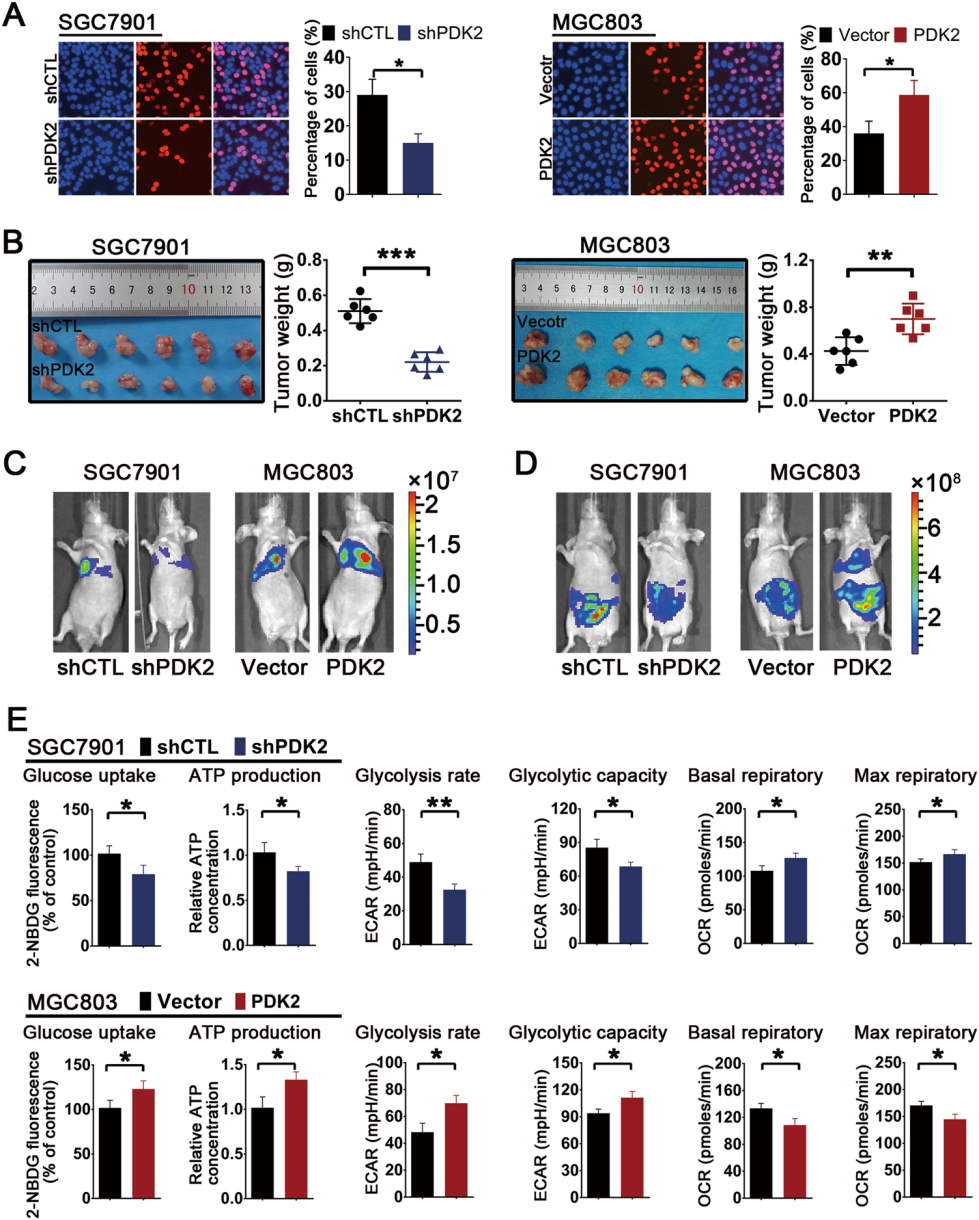
Analysis of PDK2 effects on tumor growth and glucose metabolism. **a** DNA synthesis in GC cells was measured by Edu incorporation assay. Red fluorescence indicates Edu-positive cells; blue fluorescence from the Hoechst stain represents total cells. **b** The in vivo effect of PDK2 was evaluated in xenograft mouse models bearing tumors originating from SGC7901 and MGC803 cells; *n* = 6 per group. **c** Bioluminescent images of mice administered with SGC7901 and MGC803 cells via the lateral tail veins. Images were taken after 28 days. *n* = 6 per group. **d** Bioluminescent images of the metastatic tumors in mice 28 days after intraperitoneal injection with GC cells. *n* = 6 per group. **e** Analysis of PDK2 effects on glucose metabolism. The error bars represent the mean (*n* = 3) ± S.D. *P < 0.05, **P < 0.01, ***P < 0.001 versus corresponding NC

To determine the impacts of PDK2 on GC cells, we elevated PDK2 expression in MGC803 and HGC27 cells, and repressed its expression in SGC7901 and BGC823 cells by lentivirus infection and verified the altered expression of PDK2 in these GC cell lines by western blotting (Supplementary Figure 5A). Using Edu incorporation and colony formation assays, we found that loss of PDK2 expression significantly suppressed the proliferation of SGC7901 cells. Conversely, ectopic expression of PDK2 markedly promoted the proliferation of MGC803 cells (Fig. 5a, Supplementary Figure 5B and 5C). To confirm that miR-422a suppressed proliferation and migration of GC cells through targeting PDK2, we performed the rescue experiments. As shown in Supplementary 6A and 6B, the attenuated cell proliferation and migration induced by miR-422a overexpression could be corrected by PDK2 overexpression; also, abnormal high proliferation and enhanced migration caused by miR-422a deletion could be weakened by PDK2 knockdown. Next, we evaluated the in vivo effect of PDK2 using a xenograft mouse model. Tumor volume and Ki67 expression were significantly increased in tumors derived from PDK2-overexpressing MGC803 cells, whereas opposing results were obtained after PDK2 deletion in SGC7901 cells (Fig. 5b and supplementary Figure 5D). Ectopic expression of PDK2 significantly promoted metastasis potential and peritoneal dissemination in vivo, whereas PDK2 overexpression in MGC803 cells significantly inhibited both (Figs. 5c, d). Furthermore, PDK2′s functions in cellular metabolism were analyzed and found to be opposite to those of miR-422a in GC. PDK2 knockdown in SGC7901 cells markedly decreased glucose uptake and ATP levels, whereas forced PDK2 expression in MGC803 cells had the opposite effects (Fig. 5e). Finally, loss of PDK2 expression in SGC7901 cells markedly decreased both the glycolytic capacity and the glycolysis rate of the cells, whereas PDK2 overexpression in MGC803 cells significantly enhanced both glycolytic capacity and glycolysis (Fig. 5e and Supplementary Figure 5E). The mitochondrial function of oxidative phosphorylation was also affected by PDK2 level. PDK2 deletion in SGC7901 cells resulted in a markedly enhanced basal OCR and significantly elevated maximum OCR relative to control cells. However, PDK2 overexpression in MGC803 cells led to lower OCR at both basal and maximal levels (Fig. 5e and Supplementary Figure 5F). Taken together, these results show that PDK2 promotes malignancy and the Warburg Effect in GC.

### The miR-422a–PDK2 axis modulates ROS production and the G1/S transition in GC

**Fig. 6:**
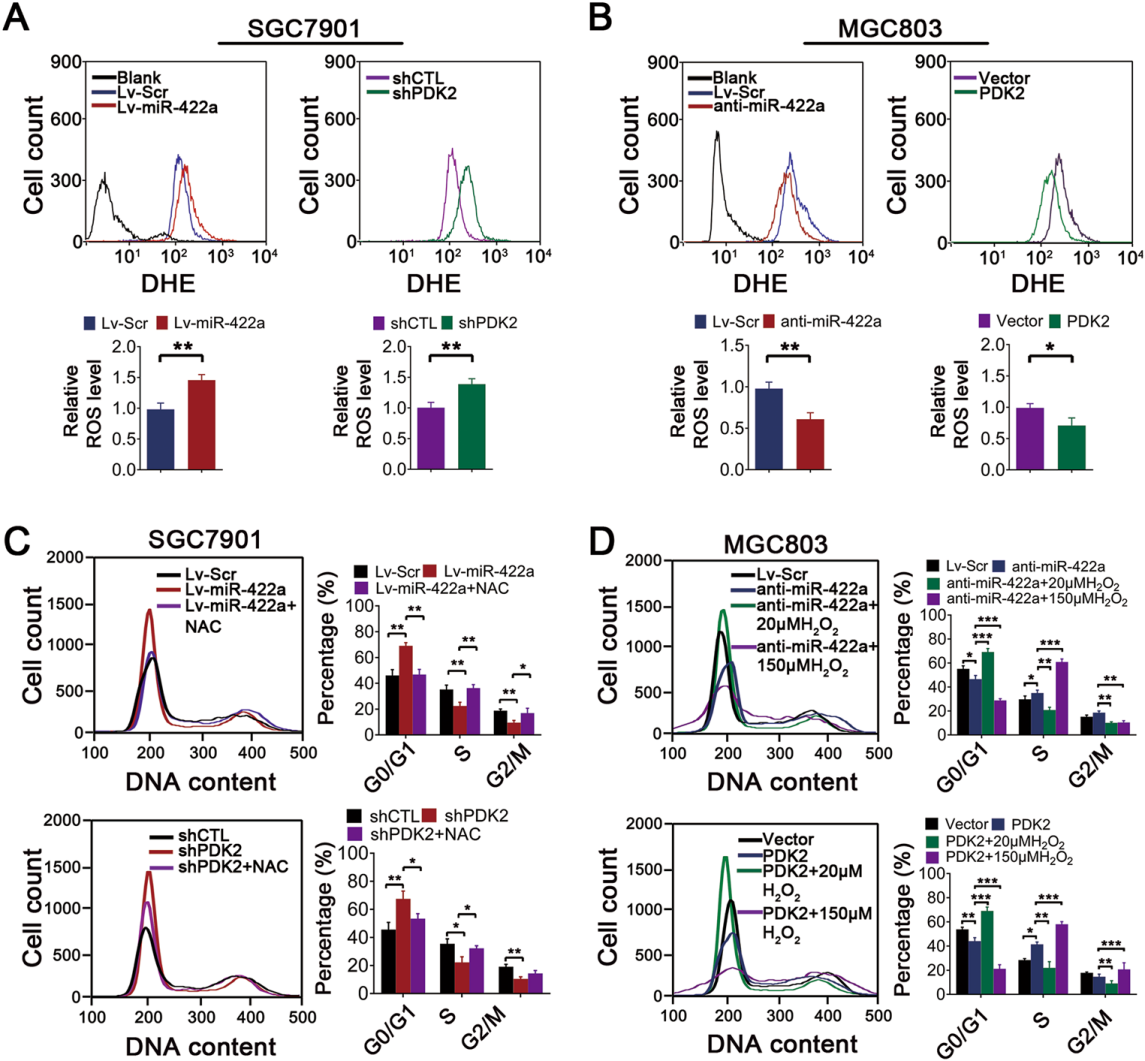
Analysis of the effects of the miR-422a–PDK2 axis on ROS production and cell cycle progression in GC. **a** FACS analysis (above) and statistical results (below) of ROS levels after miR-422a overexpression or PDK2 knockdown in SGC7901 cells. **b** FACS analysis (above) and statistical results (below) of ROS levels after miR-422a knockdown or PDK2 overexpression in MGC803 cells. **c** DNA content analysis of SGC7901 cells after transfection with Lv-miR-422a, sh-PDK2, negative control, or 10 mM NAC for 24 h as indicated. **d** DNA content analysis of MGC803 cells after transfection with anti-miR-422a, PDK2, negative control, 20 μM or 150 μM H_2_O_2_ for 24 h. The error bars represent the mean (*n* = 3) ± S.D. *P < 0.05, **P < 0.01, ***P < 0.001 versus corresponding NC

Previous studies have demonstrated that metabolic reprogramming and ROS homeostasis are intertwined in cancer cells^30,31^. As expected, miR-422a overexpression or PDK2 knockdown resulted in obvious elevation of ROS levels, whereas miR-422a downregulation or overexpression of PDK2 decreased ROS production (Figs. 6a, b, Supplementary Figure 8A and 8B). The induction of ROS has been reported to be related to tumor progression, and survival. However, we observed no significant differences in tumor cell apoptosis after miR-422a overexpression or knockdown (Supplementary Figure 7A). We further analyzed whether the miR-422a–PDK2 axis modulates cell cycle progression. As shown in Fig. 6c, miR-422a overexpression and PDK2 knockdown in SGC7901 cells significantly increased the percentage of cells in the G1 phase and decreased the percentage of cells in the S phase. However, these changes in the cell cycle profile could be rescued by treatment with the antioxidant *N*-acetylcysteine (NAC). Furthermore, miR-422a knockdown and PDK2 overexpression markedly increased the number of cells in S phase. Nevertheless, these effects could be prevented by treatment with low molar concentrations of H_2_O_2_ (20 μM). High concentrations of H_2_O_2_ (100 μM), however, resulted in obvious S phase arrest (Fig. 6d and Supplementary 8C). Taken together, these results suggest that the miR-422a–PDK2 axis regulates ROS levels and subsequently affects cell cycle progression.

### The miR-422a–PDK2–ROS axis prevents the G1/S transition by regulating the RB/E2F1 pathway

**Fig. 7:**
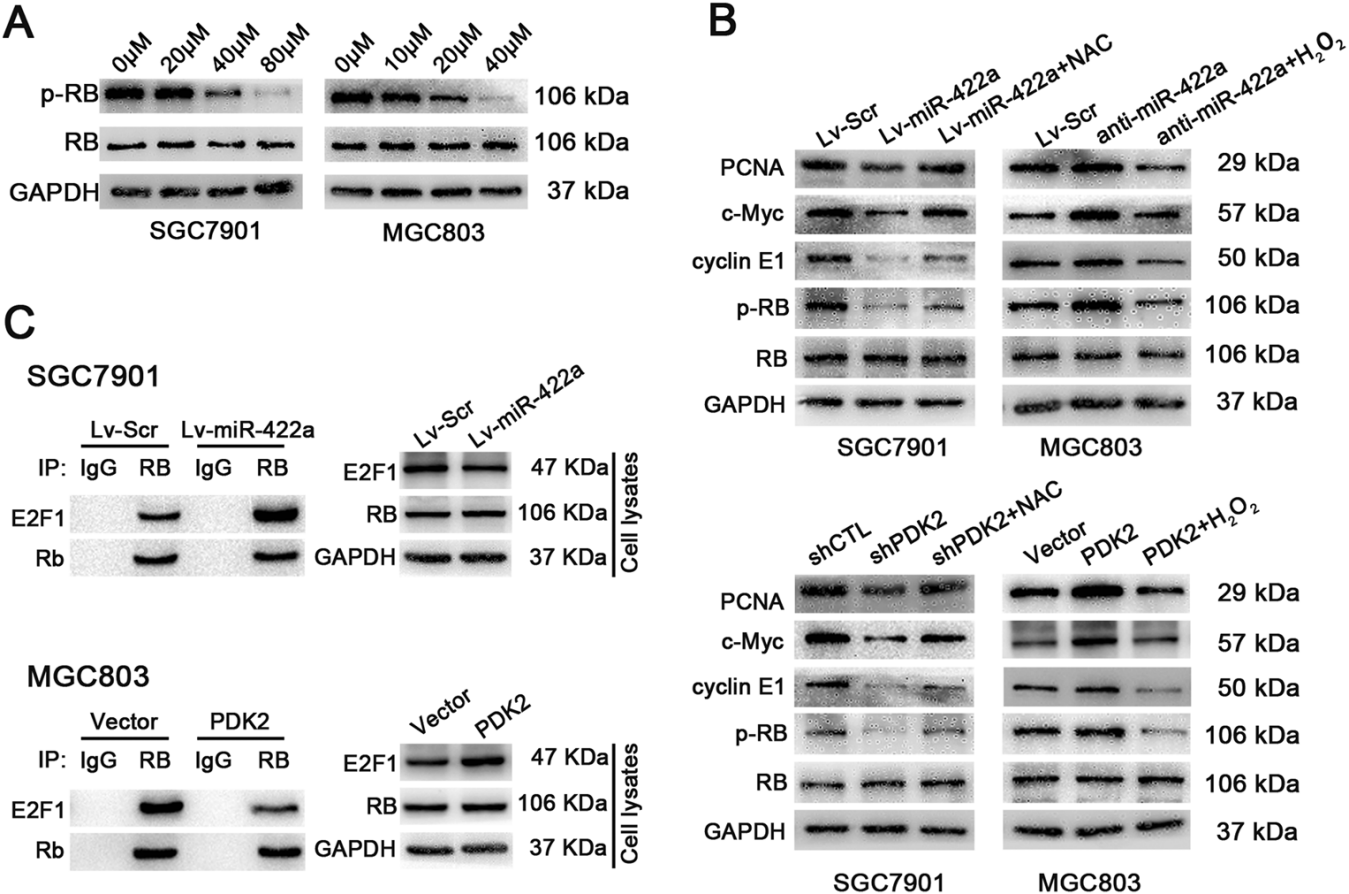
ROS inhibits the RB-E2F1 pathway. **a** H_2_O_2_ treatment causes a decrease in the levels of phosphorylated RB in SGC7901 and MGC803 cells within 24 h. **b** E2F1, RB, p-RB, cyclin E1, c-myc and PCNA were measured by western blotting after transfection with miR-422a, mock miR-422a, anti-miR-422a, PDK2 and sh-PDK2, and treatment with either 20 μM H_2_O_2_ or 10 mM NAC, as indicated. **c** Total protein extracts were subjected to IP using anti-RB antibody or control IgG, followed by western blotting with E2F1 antibody. All the experiments were repeated three times

Given that the RB/E2F1 pathway is crucial to the G1/S transition process^32^, we assumed that low concentrations of ROS would have an impact on the levels of E2F1 or RB phosphorylation. To test this hypothesis, we treated SGC7901 and MGC803 cells with H_2_O_2_ and found that low molar concentrations of H_2_O_2_ led to a dose-dependent reduction in RB phosphorylation (Fig. 7a). Similarly, miR-422a overexpression and PDK2 knockdown resulted in significant RB hypophosphorylation. However, these effects could be rescued by treatment of the cells with NAC. In contrast, miR-422a downregulation and PDK2 overexpression had effects opposite to those observed after miR-422a overexpression and PDK2 knockdown (Fig. 7b and Supplementary Figure 8C). Moreover, the expression of several E2F1 targets, including *c*-myc, cyclin E1 and proliferating cell nuclear antigen (PCNA), was inhibited by miR-422a overexpression, PDK2 downregulation and treatment with 20 μM H_2_O_2_. Conversely, miR-422a knockdown, PDK2 overexpression and treatment with 10 mM NAC elevated the levels of *c*-myc, cyclin E1 and PCNA (Fig. 7b). In addition, enhanced expression of miR-422a in SGC7901 cells promoted the formation of RB-E2F1 complexes, whereas the opposite results were obtained in MGC803 cells with miR-422a deletion (Fig. 7c). Finally, RB hypophosphorylation induced by miR-422a overexpression could be corrected by PDK2 overexpression; also, high level of RB phosphorylation caused by miR-422a deletion could be weakened by PDK2 knockdown (Supplementary Figure 6C). These results indicate that the miR-422a–PDK2–ROS axis induces G1 arrest via modulating the RB-E2F1 pathway.

### The miR-422a–PDK2 axis regulates ROS level via de novo lipogenesis

**Fig. 8:**
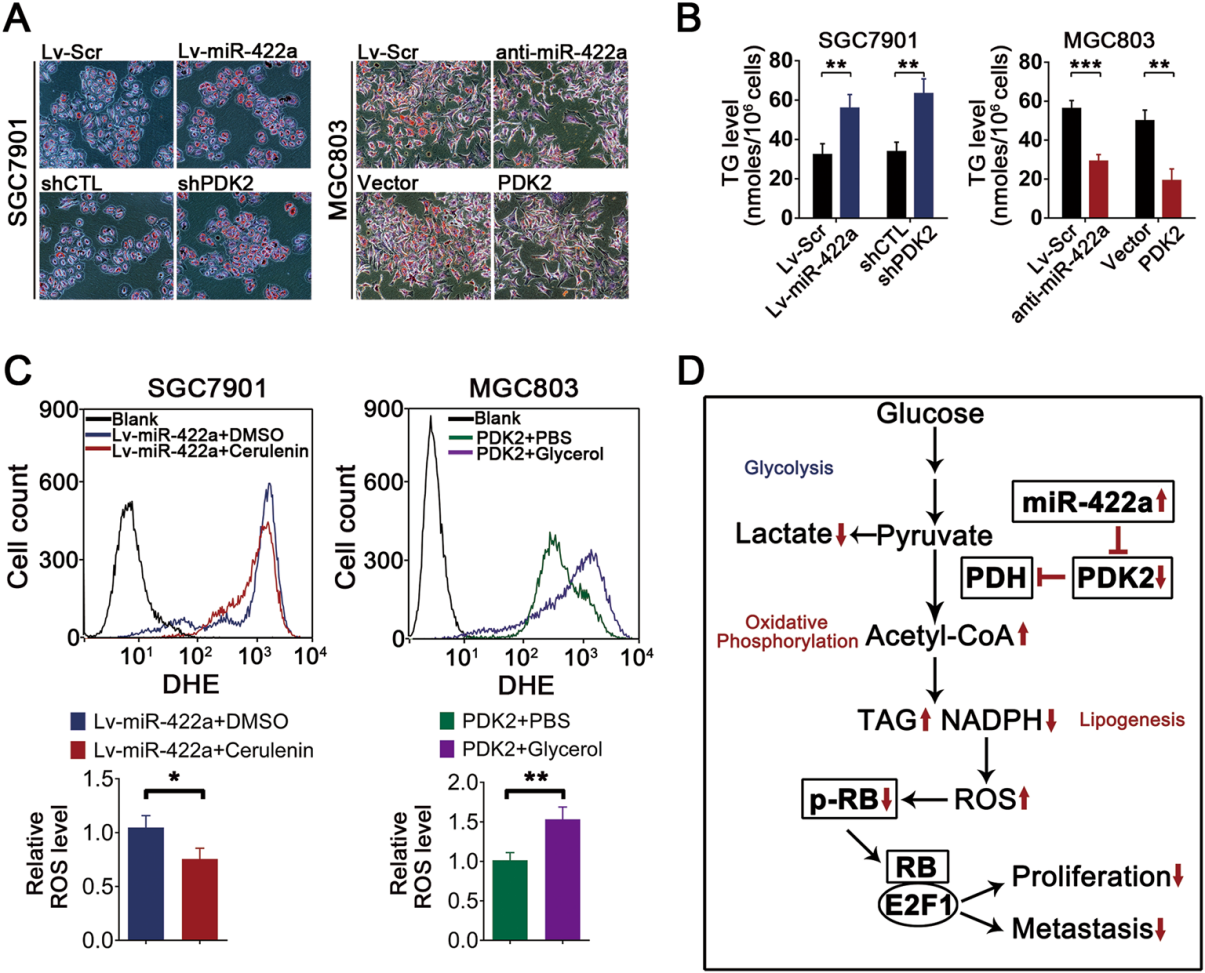
The miR-422a–PDK2 axis regulates ROS production through de novo lipogenesis. **a** Lipogenesis was measured by Oil-Red-O staining. **b** The cellular TAG concentration was quantified by employing a TAG detection kit. **c** FACS analysis (above) and statistical results (below) of ROS levels after treatment with 2 mg/L cerulenin or 0.7 mol/L Glycerol for 24 h. **d** Schematic for regulation of cellular metabolism and malignancy by the miR-422a–PDK2 axis in GC. The error bars represent the mean (*n* = 3) ± S.D. *P < 0.05, **P < 0.01, ***P < 0.001 versus corresponding NC

Because the miR-422a–PDK2 axis promotes the conversion of pyruvate into acetyl-CoA, a critical ingredient in de novo lipogenesis, we speculated that fatty acid synthesis might be influenced by manipulation of this axis. Using Oil Red O staining and Triacylglycerol (TAG) assays, we found that miR-422a overexpression and PDK2 knockdown in SGC7901 cells significantly promoted lipid generation, whereas miR-422a knockdown and PDK2 overexpression markedly impaired fatty acid synthesis (Figs. 8a, b). In the process of de novo lipogenesis, nicotinamide adenine dinucleotide phosphate (NADPH), a crucial antioxidant that provides reducing power, is consumed, thus influencing the cellular ROS level. To confirm that de novo lipogenesis mediates the effect of the miR-422a–PDK2 axis on ROS production, we treated cancer cells with cerulenin, a chemical inhibitor of the key lipogenic enzyme fatty acid synthetase (FASN), or with glycerol, an compound that promotes TAG synthesis^33^, and assessed the rescue effects of the two substances on ROS levels. As shown in Fig. 8c, cerulenin decreased ROS production in miR-422a-overexpressing SGC7901 cells, whereas glycerol increased ROS production in MGC803 cells in which PDK2 expression had been upregulated (Fig. 8c). Taken together, the data suggest the miR-422a–PDK2 axis may regulate ROS level via de novo lipogenesis.

## Discussion

MiRNAs have come to be regarded as novel diagnostic biomarkers, as potential prognostic predictors and as promising therapeutic targets of GC^7,34,35^. However, further exploration of the clinical significance, biological effects and potential mechanisms by which specific miRNAs affect GC is needed. MiR-422a is implicated in the development of several cancer types, including colorectal cancer, osteosarcoma and lung cancer^36–38^. Here, we demonstrated that miR-422a is remarkably lower in GC tissues than in matched normal tissues. Moreover, we found that miR-422 expression level was strongly related to tumor size and invasiveness and that forced miR-422a expression resulted in inhibition of GC cell proliferation both in vitro and in vivo. These observations indicate that miR-422a probably acts as a tumor suppressor.

Cancer cells preferentially utilize glycolysis rather than mitochondrial oxidative phosphorylation (OXPHOS) to produce glucose-dependent ATP and glycolytic intermediates for macromolecular biosynthesis^8,39,40^. In recent years, many studies have demonstrated that miRNAs play important roles in the metabolic reprogramming of cancer cells^12,41–43^. Based on functional studies, we hypothesized that miR-422a may exert its antitumor effects by modulating metabolic processes. In our study, PDK2 proved to be a direct functional target of miR-422a. There are four PDK isozymes, PDK1–4^44–46^. Although all these of PDKs serve the same purpose, they may have different or even opposite functions in tumorigenesis. For instance, previous studies have demonstrated that PDK1 acts as a tumor promoter by facilitating metabolic adaptation to nutrient limitation and hypoxia in liver cancer^15^, whereas PDK4 plays a tumor-suppressing role in non-small-cell lung carcinoma (NSCLC)^41^. In our study, PDK2 promotes tumor growth, elevates ATP production and facilitates the metabolic shift from oxidative phosphorylation to aerobic glycolysis in GC cells.

By phosphorylating the E1α subunit of PDH, PDKs decrease its activity and reduce the conversion of pyruvate to acetyl-CoA, resulting in the switching of carbon flow from the tricarboxylic acid cycle (TCA) cycle and de novo lipogenesis to lactate production. Hence, the miR-422a–PDK2 axis may convert additional pyruvate to acetyl-CoA, thus fueling the TCA cycle and lipogenesis. Previous studies have demonstrated that excessive oxidative phosphorylation may increase ROS production^47,48^. On the other hand, one of the most important substrates of de novo lipogenesis is NADPH, a crucial antioxidant that provides reducing power to the glutathione and thioredoxin systems^49^, the dysregulation of which may influence the cellular ROS level. In the present study, we demonstrated that the miR-422a–PDK2 axis regulates GC cell lipogenesis and ROS levels, resulting in G1 phase arrest, rather than apoptosis of cancer cells. To further explore the mechanism involved in this effect, we postulated that the tumor-suppressor RB might be a downstream effector protein of ROS. RB restricts the ability of cancer cells to replicate by preventing their progression from the G1 to the S phase of the cell cycle^50^. When it is time for a cell to enter S phase, complexes of cyclin-dependent kinase and cyclins phosphorylate RB to p-RB, allowing E2F1 to dissociate from RB and become active^51^. Free E2F1 activates factors such as cyclins (e.g., cyclin E and cyclin A), which cause the cell to progress through the cell cycle by activating cyclin-dependent kinases and a molecule called PCNA, which increases the rate of DNA replication and repair by helping DNA polymerase attach to DNA^52–54^. Here, we showed that low concentrations of ROS leaded to a dose-dependent reduction in RB phosphorylation. Thus, our study demonstrates an essential role of ROS in the metabolic regulation of cell proliferation and thus highlights a key role of ROS in the integration of metabolism and cell proliferation.

In conclusion, we demonstrated that miR-422a and PDK2 play critical roles in GC cell progression and metabolism. The miR-422a–PDK2 axis inhibits tumor progression mainly through metabolic reprogramming. In this process, the ROS level and the RB/E2F1 pathway are also significantly affected. These observations provide new evidence for an interplay between cancer-associated signaling pathways and cell metabolism. The impact of miR-422a and PDK2 on ROS levels could be partially abrogated via modulation of de novo lipogenesis, emphasizing the crucial effects of reprogramming metabolism, not only aerobic glycolysis but also other metabolic pathways, including lipogenesis, in tumorigenesis and tumor progression. Because cancer cells must balance various metabolic pathways to address the biosynthetic needs of fast-growing tumors, a better therapeutic strategy might be combined therapy that targets multiple metabolic processes.

## Materials and methods

### Clinical samples

Primary GC samples and corresponding normal tumor adjacent specimens were obtained from 60 patients who underwent radical resection for GC at the Jiangsu Province Hospital, China, between April 2015 and February 2016. No patients suffered from uncontrollable systemic disease, secondary and recurrent tumors. No special treatment for GC was administered before surgery. For the use of these tissue specimens for research purposes, prior consent from the patients and approval from the Ethics Committee of the Jiangsu Province Hospital were obtained. In all cases, diagnoses and grading were confirmed by two experienced pathologists based on the 2010 GC staging system of the AJCC.

### Cell culture and lentivirus transfection

The human GC cell lines and the GES-1 cell line were obtained from the Shanghai Institutes for Biological Sciences, Chinese Academy of Sciences. All cell lines were cultured in RPMI-1640 (Gibco, USA) containing 10% fetal bovine serum (Gibco, Uruguay) and incubated in a humidified chamber with 5% CO_2_ at 37 °C.

Lentiviral vectors expressing miR-422a, anti-miR-422a or PDK2 short hairpin RNA (shRNA) were constructed by cloning mature miR-422a, anti-miR-422a or PDK2 shRNA fragments into the LV12- u6/Luciferase05/Puro vector (Genepharma, China). The target sequence of PDK2 shRNA was 5′-GAGGAAGATTGAGCGACTCTT-3′. Stable cell lines overexpressing PDK2 were established by lentiviral transduction using the LV13-EF1a/Luciferase05/Puro vector (Genepharma, China) carrying the PDK2 DNA sequence. Stable cells were selected using puromycin.

### Antibodies and reagents

The following antibodies were used: PDK2 (ab68164), PDH-E1α (ab168379), phospho-PDH-E1α (Serine 293) (ab177461), E2F1 (ab179445), PCNA (ab29), cyclin E1 (ab3927), GAPDH (ab8245), β-actin (ab8226), Ki-67 (ab156956), horseradish peroxidase (HRP)-linked anti-rabbit IgG (ab6721), HRP-linked anti-rabbit IgG (ab6789) from Abcam, RB (#2443), RB (Serine 807/811) (#9308), c-myc (#13987) from Cell Signaling Technology.

The following reagents were used: 5-Aza (A3656,), TSA (T1952), NAC (A9165), H_2_O_2_ (88597), glycerol (G2025) from Sigma-Aldrich, cerulenin (A0210) from MedChem Express.

### RNA extraction and qRT-PCR

Total RNA was extracted from frozen tissues and cell lines using an miRNeasy kit (15596018, Invitrogen). RNA quality and concentration were measured using a NanoDrop spectrophotometer (ND-100, Thermo). miRNA reverse transcription was performed using a New Poly(A) Tailing Kit (ThermoFisher Scientific). mRNA was reverse transcribed into complementary DNA (cDNA) using PrimeScript RT Master Mix Kit (RR036A, TaKaRa). cDNA was amplified using Universal SYBR Green Master Mix (4913914001, Roche). The β-actin and U6 genes were employed as internal controls for gene and miRNA expression, respectively. The sequences of the primers used in this study are listed in Supplementary Table 1.

### Fluorescence in situ hybridization

MiR-422a expression in GC and adjacent normal tissues was detected by FISH. The mature human miR-422a sequence is 5′-ACUGGACUUAGGGUCAGAAGGC -3′. We used LNA (locked-nucleic acid)-based probes directed against the full-length mature miRNA sequence. The 5′-FAM-labeled miR-422a probe sequence was 5′-GCCTTCTGACCCTAAGTCCAGT-3′; the probe was purchased from BioSense (Guangzhou, China). The FISH assay was performed as previously described^55^.

### Cell proliferation and colony formation assay

Cell proliferation was measured using the CCK-8 assay (CK04, Dojindo). Cells were seeded in triplicate in 96-well plates at a density of 2000 cells per well and cultured in RPMI-1640 supplemented with 10% FBS for 6 days. Subsequently, 10 μl of CCK-8 solution was added to each well, and the plates were incubated for 2 h. The absorbance (OD) at 450 nm was then determined. For colony formation assays, cells were seeded in six-well plates at a density of 500 cells per well and incubated for 2 weeks. The colonies were fixed with 75% alcohol and stained with crystal violet. Colonies containing >50 cells were counted under a microscope.

### EdU incorporation assay

Each group of isolated tumor cells was seeded in 24-well plates in triplicate at a density of 5 × 10^4^ cells/well and incubated for 48 h. Subsequently, the cells were incubated for an additional 2 h in medium containing 50 μM EdU (C00052, RiboBio). Cell proliferation was measured using a Cell-Light™ EdU Cell Proliferation Detection Kit (C10301, RiboBio) according to the manufacturer’s instructions. DNA was stained with Hoechst 33342 (300 μl/well) for 30 min and visualized using an inverted fluorescence microscope. For each EdU experiment, five random fields were imaged at 100× magnification. The number of EdU-positive cells was determined by Hoechst nuclear staining and was expressed as a percentage of the total number of cells in each field.

### Cell migration assay

Cell migration was measured using Transwell plates (Corning) according to the manufacturer’s instructions using 10% FBS as a chemoattractant. After seeding 3 × 10^4^ cells in the top chambers of the plates, the plates were incubated for 16 h. Subsequently, cells on the top side of the membrane were washed off; migrating cells were stained with crystal violet and counted under a microscope.

### Glucose uptake, lactate and ATP assay

For glucose uptake assays, cells were incubated with 100 μM 2-NBDG (11046, Cayman) beginning 30 min before the end of the experimental time. The cells were then washed with phosphate-buffered saline (PBS), resuspended in ice-cold PBS, collected and analyzed cytofluorometrically by recording FL-1 fluorescence. For analysis of lactate concentration, the indicated cells were cultured for 48 h. Whole-cell lysis was then determined using a lactate assay kit (K627, BioVision) according to the manufacturer’s instructions. For measurement of ATP levels, whole-cell extracts from the indicated cells were lysed in the lysis buffer provided an ATP assay kit (S0026, Beyotime). Intracellular ATP was evaluated by luciferase activity according to the standard protocol described in the ATP assay kit.

### OCR/ECAR measurements

Glycolytic capacity and cellular mitochondrial function were determined using a Seahorse XF24 analyzer (Seahorse Biosciences) according to the manufacturer’s instructions. Briefly, 2 × 10^5^ cells were seeded in Seahorse plates, incubated overnight and washed in Seahorse buffer. Then, 175 μl of Seahorse buffer containing 25 μl each of 1 μmol/L oligomycin, 1 μmol/L FCCP and 1 μmol/L rotenone was added to measure the OCR. To determine the ECAR, 175 μl of Seahorse buffer and 25 μl each of 10 mmol/L glucose, 1 μmol/L oligomycin and 100 mmol/L 2-DG were automatically injected into the analyzer.

### Western blotting and co-immunoprecipitation

Total protein was prepared using a protein extraction kit (KGP9100, Key Gene). Equal amounts of protein were separated on 10% gels by sodium dodecyl sulfate–polyacrylamide gel electrophoresis and transferred to nitrocellulose membranes. After blocking in a mixture of Tris-buffered saline and Tween-20 (TBST) containing 5% bovine serum albumin (BSA), the membranes were incubated with specific primary antibodies followed by secondary antibodies. The signals were detected using the Chemiluminescence HRP Substrate (WBKL0100, Millipore) and an enhanced chemiluminescence detection system. Co-immunoprecipitations were performed using the Dynabeads Protein G Immunoprecipitation Kit (Life Technologies) following the manufacturer's instructions. Negative controls were performed on all runs using an equivalent concentration of a subclass-matched immunoglobulin.

### Luciferase dual-reporter assays

The 3′-UTRs of PDK1 and PDK2 containing putative miR-422a target regions were cloned into a firefly luciferase reporter, the pmirGLO Dual-luciferase vector (GenePharma, China). In addition, the mutant miR-422a putative target regions were generated using the same approach. SGC7901 cells overexpressing miR-422a or its control were transfected with PDK1–3′-UTR-Wt, PDK1–3′-UTR-Mut, PDK2–3′-UTR-Wt or PDK2–3′-UTR-Mut, lysed 48 h after transfection and assayed using a Dual-Luciferase Reporter Assay System (E1910, Promega).

### Cell cycle, apoptosis and ROS level analyses

Cell cycle analysis was conducted by fluorescence-activated cell sorter (FACS) of >10,000 cells stained with propidium iodide (PI). For the analysis of apoptosis, cells were stained using a PE Annexin V Apoptosis Detection Kit I (559763, BD Pharmingen) according to the manufacturer’s instructions, and detected by FACS. Intracellular ROS levels were examined by FACS of at least 10,000 cells that had been stained with DHE (D7008, Sigma).

### Animal studies

For tumor growth assay, a total of 2 × 10^6^ logarithmically growing GC cells transfected with miR-422a, anti-miR-422a, PDK2 and sh-PDK2 or the control (*N* = 6 per group) in 100 μl PBS were subcutaneously injected into the flanks of 4-week-old female nude mice. After 30 days, the resulting tumors were excised and fixed in 4% paraformaldehyde for immunohistochemical analysis of the Ki-67 expression.

For tumor metastasis assay, a total of 1 × 10^6^ logarithmically growing GC cells transfected with miR-422a, anti-miR-422a, PDK2 and sh-PDK2 or the control (*N* = 6 per group) in 100 μl PBS were injected into the lateral tail veins of nude mice or intraperitoneally injected. After 4 weeks, the IVIS Imaging system (Caliper life Sciences, USA) was used to observe the occurrence of metastases. Care of experimental animals was in accordance with Nanjing Medical University Institutional Animal Care and Use Committee.

### Immunochemical staining

All specimens used for immunochemical staining were fixed in 4% formalin and embedded in paraffin. The paraffin mass was cut into 4-μm sections that were mounted on slides and incubated with ki-67 antibody at 4 °C overnight. The slides were then washed three times with PBS and incubated with HRP-polymer-conjugated secondary antibody at room temperature for 1 h. Finally, the slides were stained with a 3,3-diaminobenzidine solution for 3 min and counterstained with haematoxylin. The slides were examined in a blinded manner. Three fields on each slide were selected for examination, and the percentage of positive tumor cells and the cell-staining intensity in these fields were determined.

### DNA methylation analysis

Genomic DNA from GES-1, BGC823 and SGC7901 GC cell lines was modified with bisulfite, and the CpG islands were amplified by PCR. The PCR products were separated by agarose gel electrophoresis (3%), extracted and cloned into the pUC18 T-vector (Sango, Shanghai). After bacterial amplification of the cloned PCR fragments by standard procedures, 10 clones were sent for DNA sequencing (Sango, Shanghai).

### Oil Red O staining and cellular TAG measurement

For Oil Red O staining, cells were seeded in six-well plates. After overnight incubation, the cells were treated with Oil Red O (MAK194, Sigma) to assess lipid metabolism. Oil Red O staining was performed according to the manufacturer’s instructions. For cellular TAG measurement, cells were collected and lysed, and a TAG detection kit (E1013, Applygen) was used to determine the TAG level. The TAG levels of the different groups were normalized to the amount of cellular protein in each group.

### Statistical analysis

All statistical analyses were performed using SPSS 20.0 software (SPSS Inc., Chicago, IL, USA); data are expressed as the mean ± S.D. *P* < 0.05 was considered statistically significant. The relative quantification of gene expression detected by real-time PCR was log-2 transformed and analyzed using Student’s *t*-test. Linear or rank correlation analysis was performed to determine correlations between gene expression levels. χ^2^ Test was used to analyze the associations of miR-422a expression with clinicopathologic features. Survival analysis was performed using the Kaplan–Meier method, and the optimal cut-off values of miR-422a relative expression were determined using X-tile software (0.05 with a *P*-value 0.0036). The data obtained in cell line experiments and animal assays were subjected to a two-tailed Student’s *t*-test or one-way analysis of variance (ANOVA; *t*-test for two-group comparisons; otherwise one-way ANOVA).

## Electronic supplementary material

Supplementary Figures

Supplementary Table 1
