# Supplementary material for: MiR-422a regulates cellular metabolism and malignancy by targeting pyruvate dehydrogenase kinase 2 in gastric cancer
Source: Cell Death Dis. 2018 May 2;9(5):505. doi: 10.1038/s41419-018-0564-3 (PMC5938701; doi:10.1038/s41419-018-0564-3)
Supplement: Supplementary file 1 — Supplementary Figures [file 41419_2018_564_MOESM1_ESM.docx]

**Supplementary Figures**

**Supplementary Figure S1**


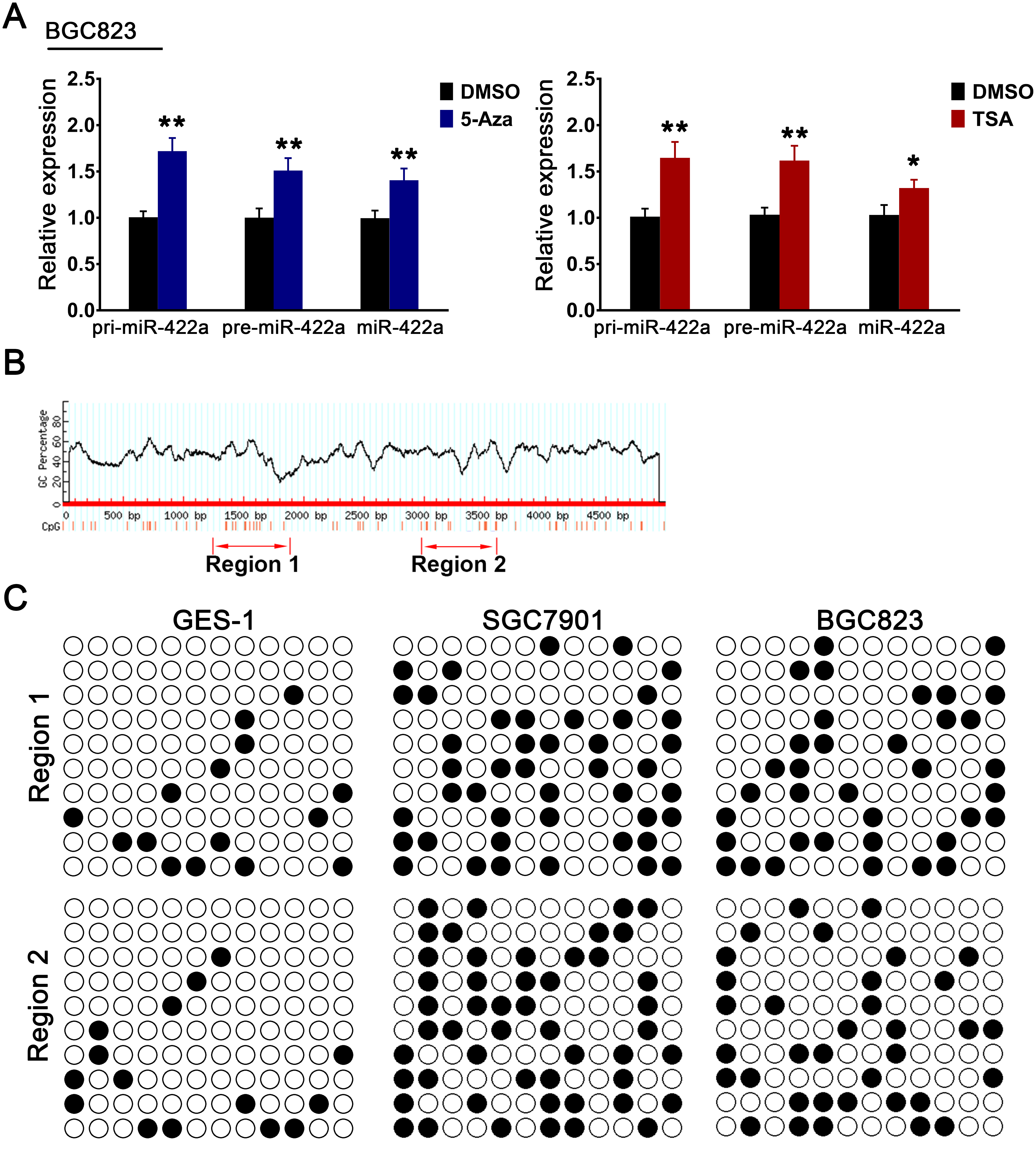


**Figure S1** **(A)** After incubation with 2.5μM 5-aza-2’-deoxycytidine (5-Aza) for 72h or with 100ng/ml trichostatin A (TSA) for 12h, the relative expression levels of pri-miR-422a, pre-miR-422a, and mature miR-422a in BCG823 cells were measured by RT-PCR. GAPDH and U6 were used for the normalization of pri-/ pre-miR and mature miR, respectively. **(B)** Scheme for the location of the CpG sites in the transcription start region of miR-422a. **(C)** The bisulfate sequencing method was used to detected the methylation of predicted CpG sites in the GES-1, SGC7901 and BGC823 cell lines. Ten clones from each cell line were analyzed. The error bars represent the mean (n=3) ± S.D. * *P* < 0.05, ** *P* < 0.01, ****P* < 0.001 versus corresponding NC.

**Supplementary Figure S2**


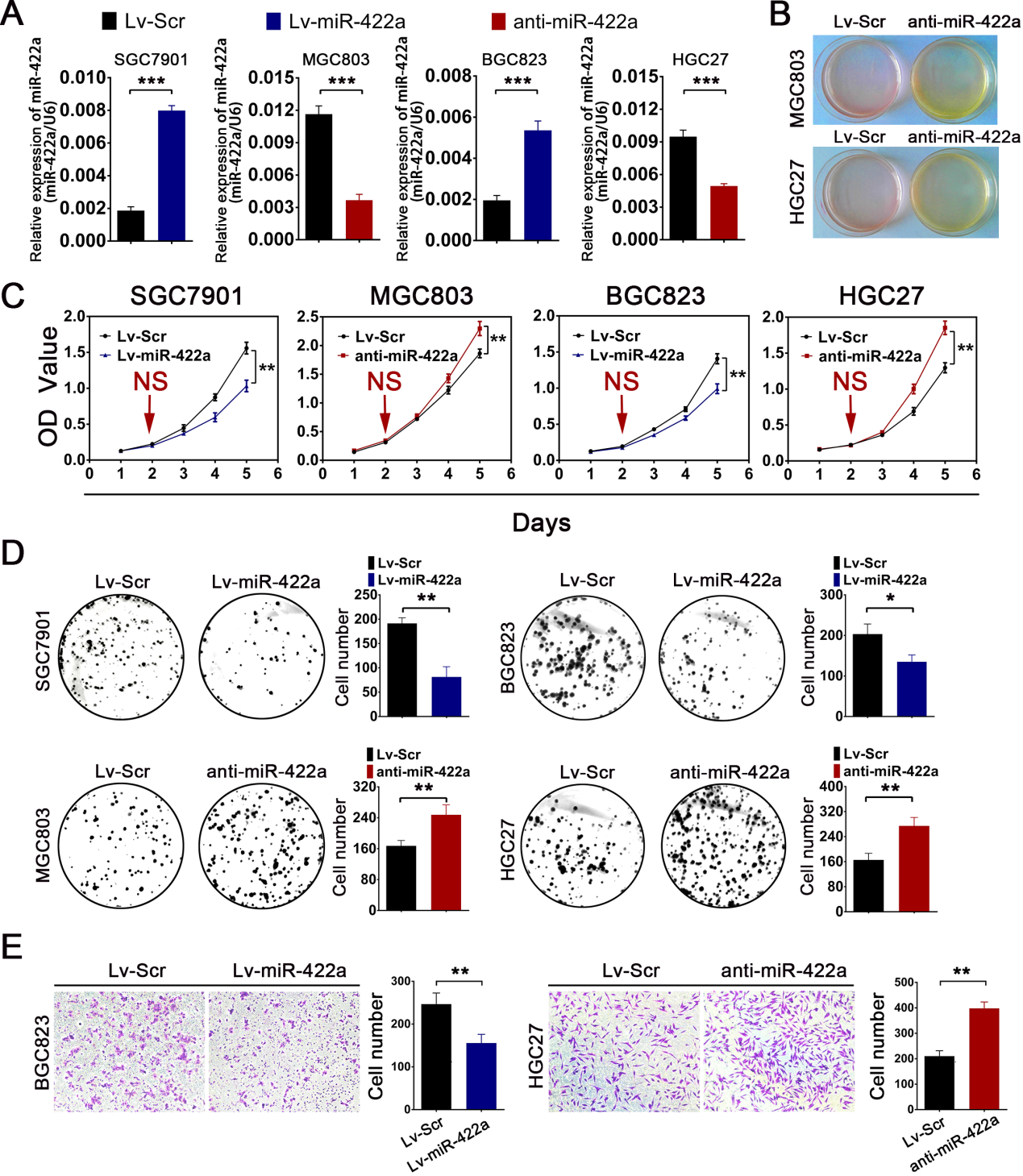


**Figure S2 (A)** miR-422a expression levels were evaluated by qRT-PCR after miR-422 overexpression and knockdown in GC cells. **(B)** Color of the culture medium after miR-422a knockdown in MGC803 and HGC27 cells. **(C)** Growth curves were recorded for 6 days, and the OD values were measured using a microplate reader at the recommended wavelength. **(D)** Colony formation by GC cells with miR-422a overexpression or knockdown. **(E)** Transwell assays were performed to evaluate the effect of miR-422a on cell migration. Cells were counted under a microscope in five randomly selected fields. Error bars represent mean (n=3) ± S.D. * P < 0.05, ** P < 0.01, P < 0.001 versus corresponding NC. NS, not significant.

**Supplementary Figure S3**


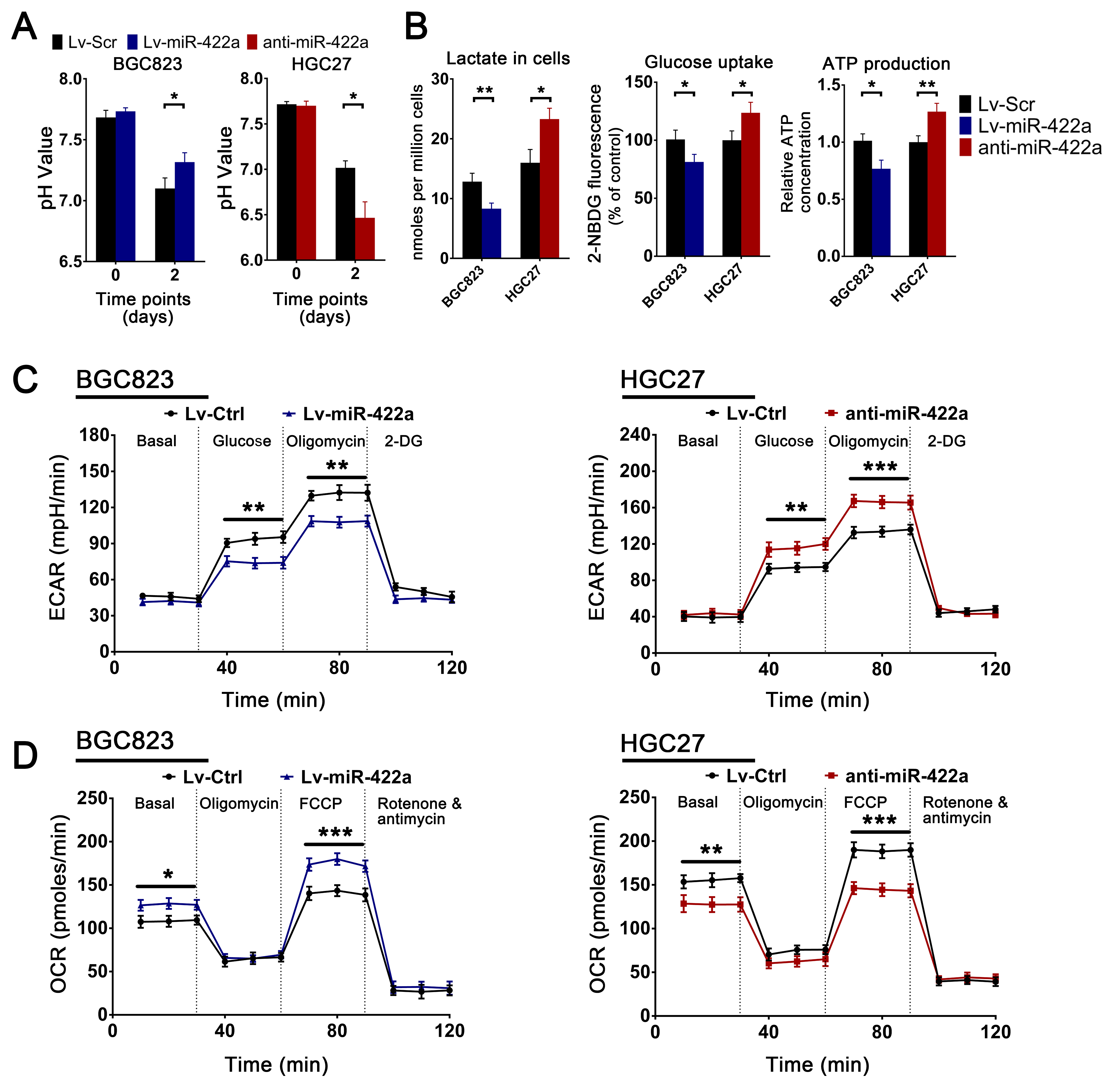


**Figure S3 (A)** The pH of the culture medium was measured at the indicated time points (day 0 and day 2). **(B)** Lactate, ATP production and glucose uptake analysis of GC cell lines were determined via flow cytometry and the colorimetric method, respectively. **(C)** Analysis of extracellular acidification rate of miR-422a-overexpressing BGC823 cells (left) and HGC27 cells with miR-422a knockdown (right). **(D)** Analysis of oxygen consumption rate of miR-422a-overexpressing BGC823 cells (left) and HGC27 cells with miR-422a knockdown (right). The error bars represent the mean (n=3) ± S.D. * *P* < 0.05, ** *P* < 0.01, ****P* < 0.001 versus corresponding NC.

**Supplementary Figure S4**


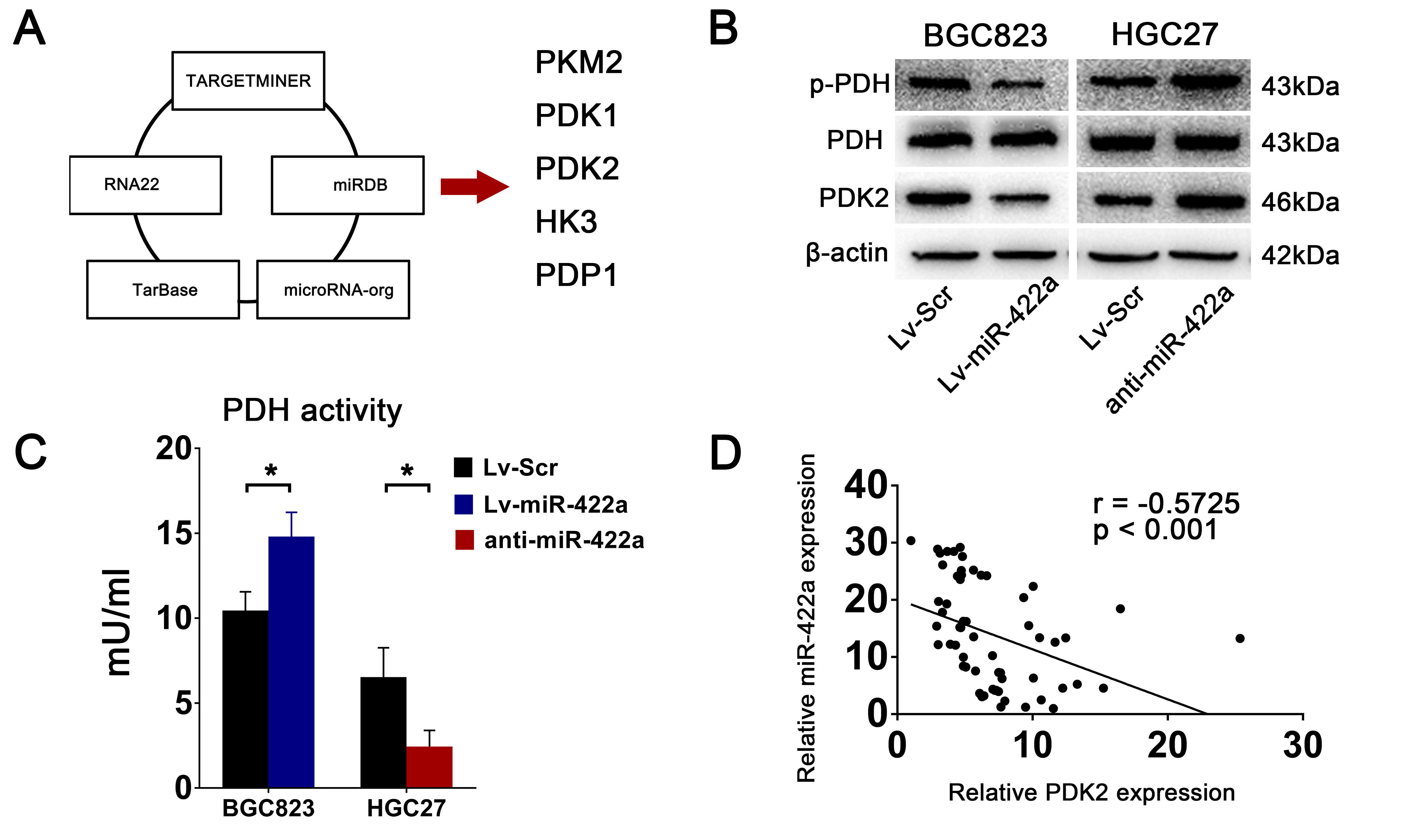


**Figure S4 (A)** Five algorithms were applied to investigate putative targets of miR-422a. **(B)** Levels of PDK2, total PDH and phosphorylated PDH E1α protein in cancer cells with miR-422a overexpression or knockdown. **(C)** After miR-422a overexpression in BGC823 and miR-422a knockdown in HGC27 cells, the cells were harvested for measurement of cellular PDH activity. **(D)** Expression correlation analysis for miR-422 and PDK2 in GC tumor samples. The error bars represent the mean (n=3) ± S.D. * *P* < 0.05, ** *P* < 0.01, ****P* < 0.001 versus corresponding NC.

**Supplementary Figure S5**


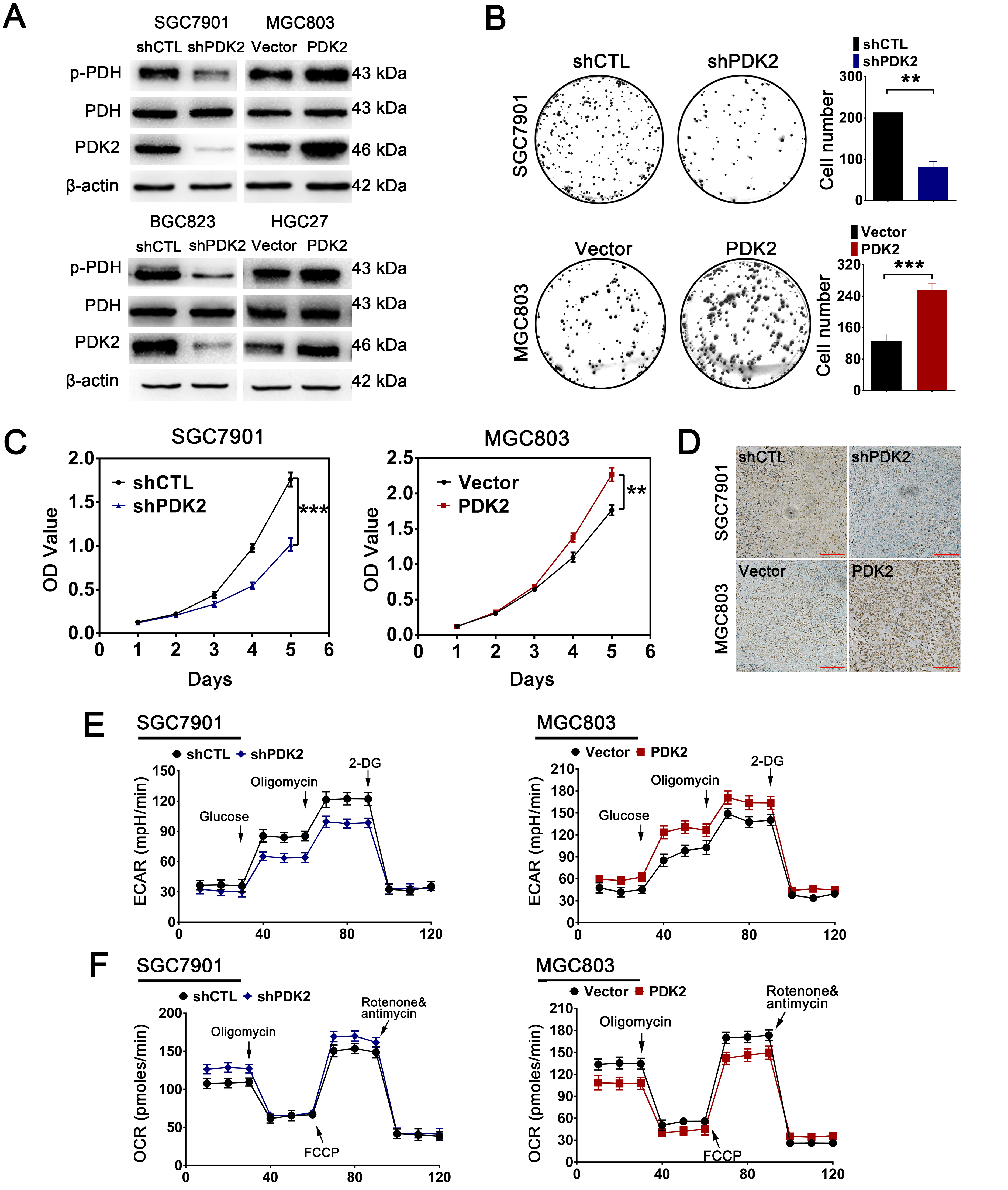


**Figure S5 (A)** PDK2 expression levels were evaluated by western blotting after PDK2 knockdown or overexpression. **(B)** Colony formation by GC cells with PDK2 overexpression or knockdown. **(C)** Growth curves were recorded for 6 days, and the OD values were measured using a microplate reader at the recommended wavelength. **(D)** Representative Ki67 staining of primary tumor tissues. Scale bar: 50 μm. **(E)** Analysis of extracellular acidification rate of SGC7901 cells with PDK2 knockdown (left) and MGC803 cells with PDK2 overexpression (right). **(F)** Analysis of oxygen consumption rate of SGC7901 cells with PDK2 knockdown (left) and MGC803 cells with PDK2 overexpression (right). The error bars represent the mean (n=3) ± S.D. * *P* < 0.05, ** *P* < 0.01, ****P* < 0.001 versus corresponding NC.

**Supplementary Figure S6**


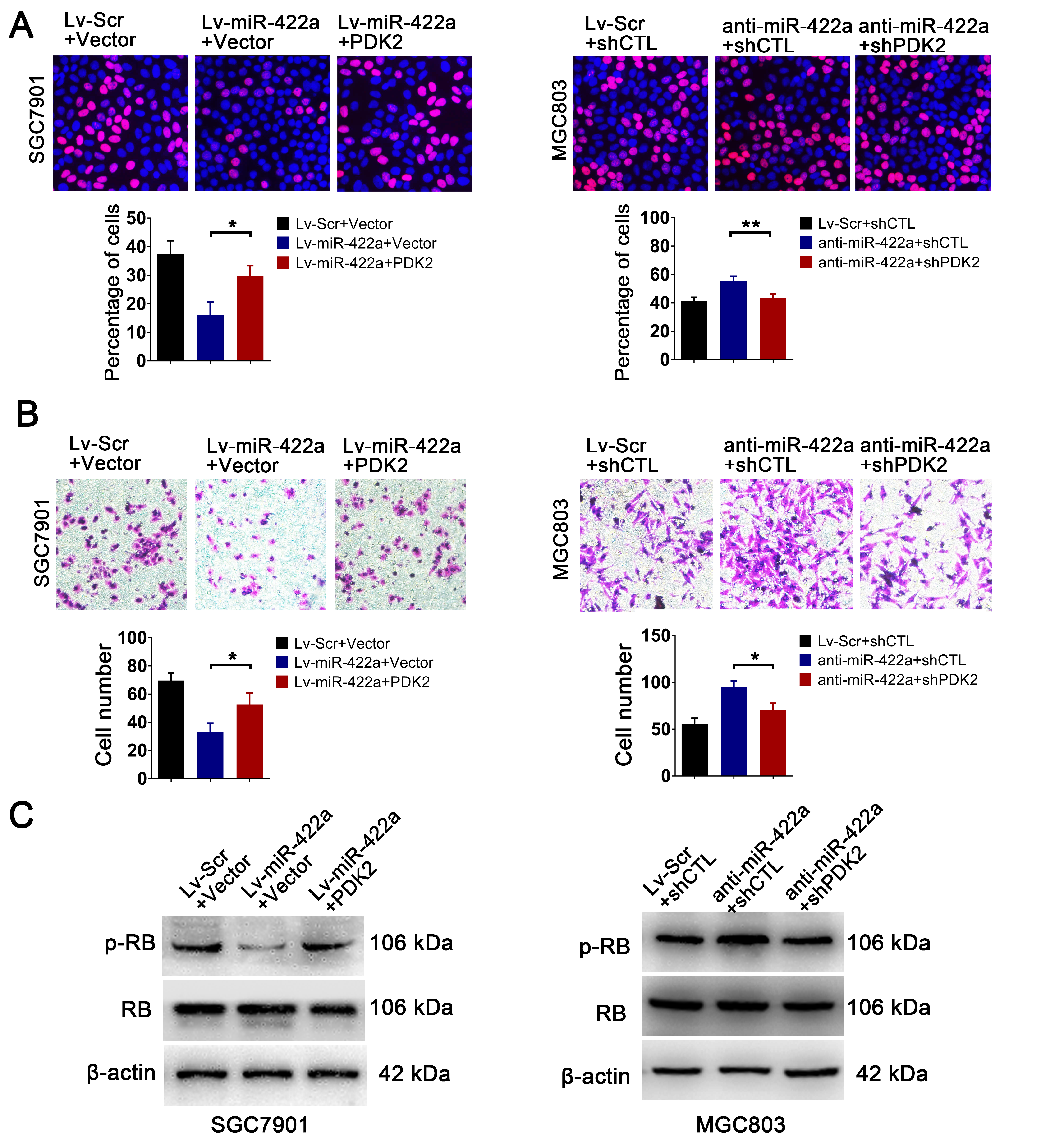


**Figure S6 (A)** DNA synthesis in GC cells was measured using the Edu incorporation assay. Red fluorescence represents Edu-positive cells; blue fluorescence from Hoechst stain represents the total cells. **(B)** Transwell assays were performed to evaluate cell migration ability. Cells were counted under a microscope in five randomly selected fields. **(C)** RB and p-RB were measured by western blotting after miR-422a overexpression with PDK2 overexpression or miR-422a deletion with PDK2 knockdown, as indicated. The error bars represent the mean (n=3) ± S.D. * *P* < 0.05, ** *P* < 0.01, ****P* < 0.001 versus corresponding NC.

**Supplementary Figure S7**


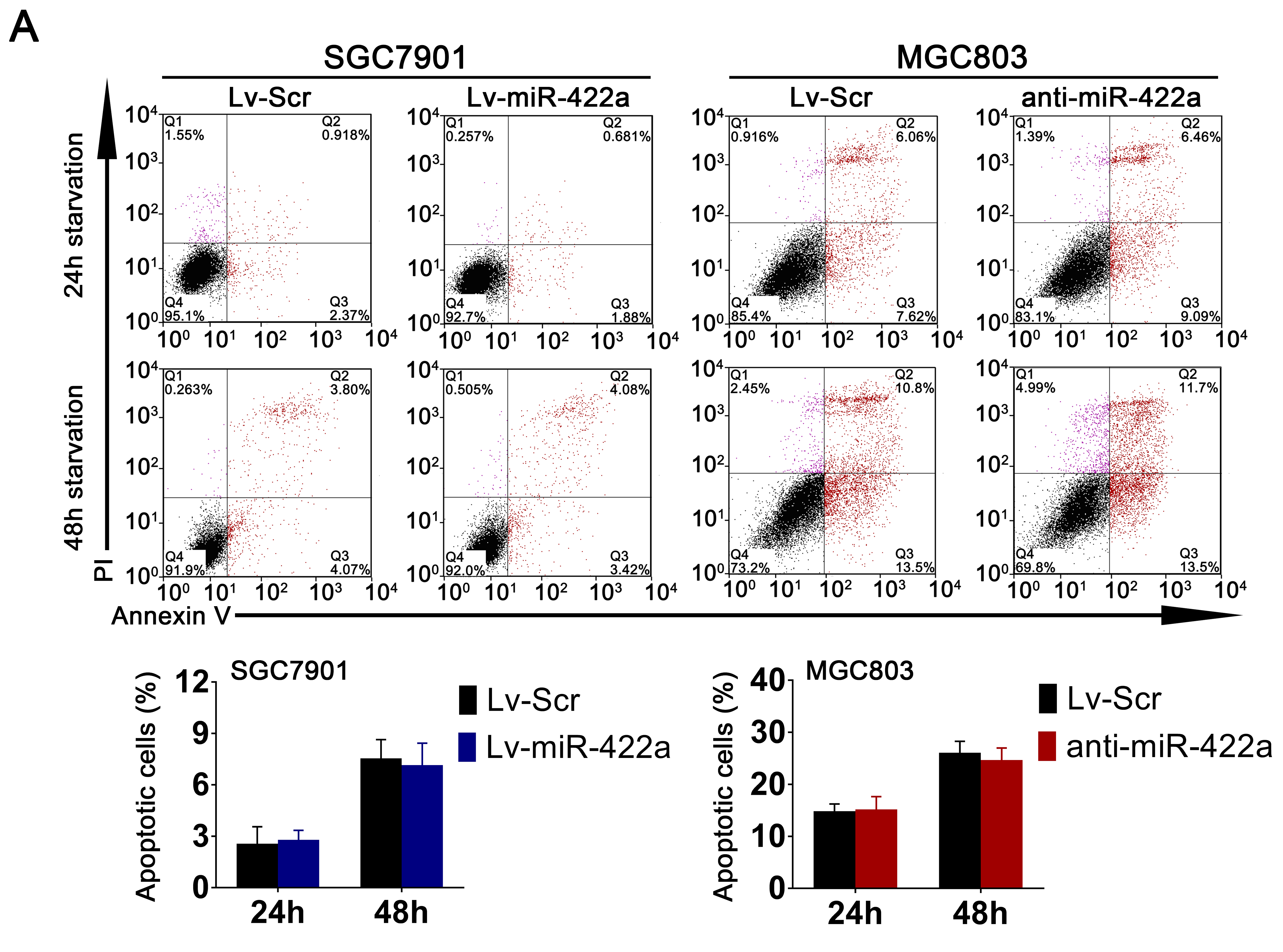


**Figure S7 (A)** Cell apoptosis was induced as previously described. GC cell lines were cultured in serum-free medium for 24h or 48h before apoptosis detection. The error bars represent the mean (n=3) ± S.D. * *P* < 0.05, ** *P* < 0.01, ****P* < 0.001 versus corresponding NC.

**Supplementary Figure S8**


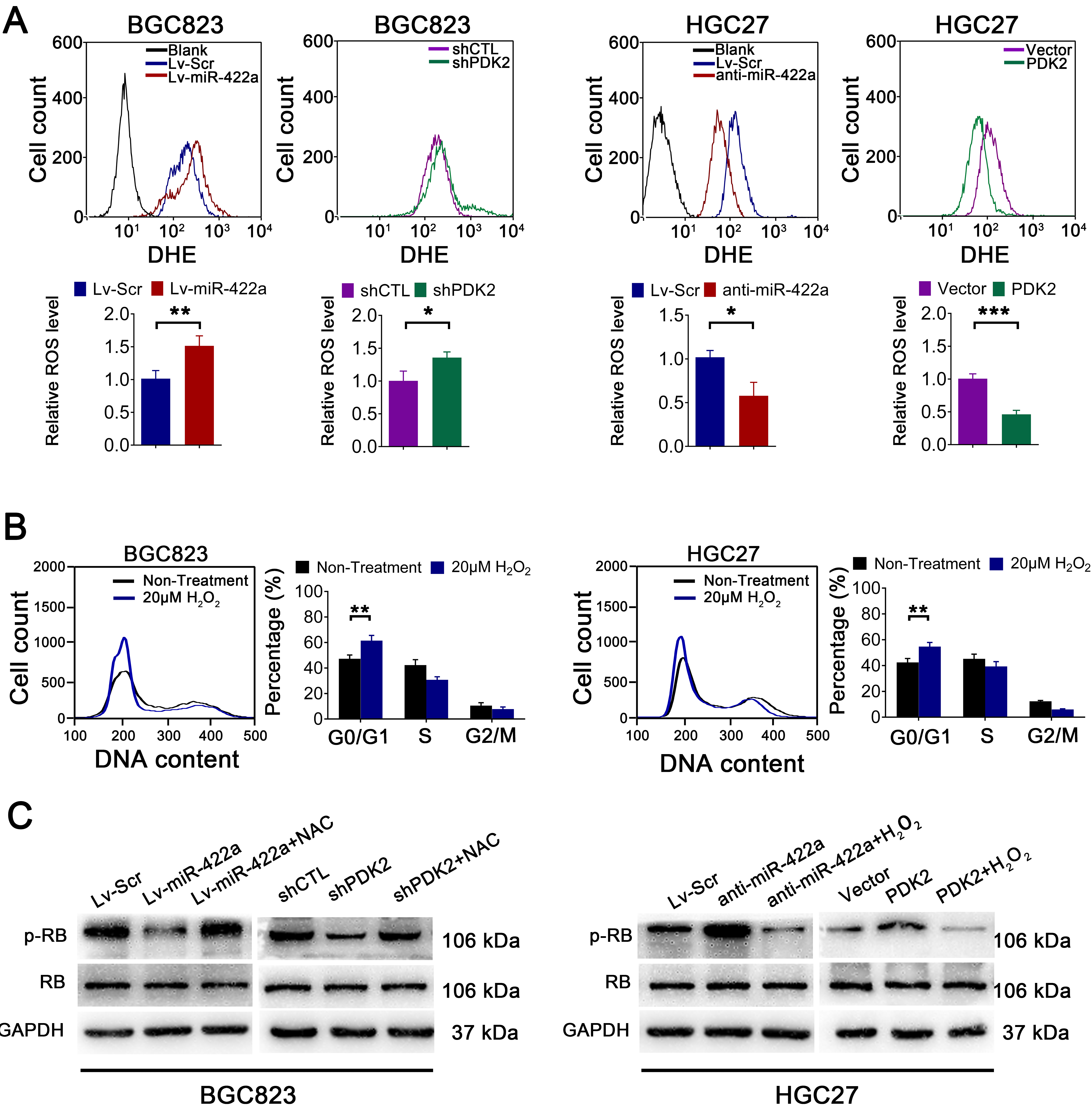


**Figure S8 (A)** FACS analysis (above) and statistical results (below) of ROS levels after altering expression of miR-422a and PDK2, as indicated. **(B)** DNA content analysis of BGC823 and HGC27 cells after treating with 20μM H_2_O_2_. **(C)** RB and p-RB were measured by western blotting after transfection with miR-422a, mock miR-422a, anti-miR-422a, PDK2, and sh-PDK2, and treatment with either 20 μM H_2_O_2_ or 10 mM NAC, as indicated., The error bars represent the mean (n=3) ± S.D. * *P* < 0.05, ** *P* < 0.01, ****P* < 0.001 versus corresponding NC.
