# Supplementary material for: MiR-422a regulates cellular metabolism and malignancy by targeting pyruvate dehydrogenase kinase 2 in gastric cancer
Source: Cell Death Dis. 2018 May 2;9(5):505. doi: 10.1038/s41419-018-0564-3 (PMC5938701; doi:10.1038/s41419-018-0564-3)
Supplement: Supplementary file 2 — Supplementary Table 1 [file 41419_2018_564_MOESM2_ESM.docx]

**Supplementary Table 1**

| miR-422a | ACTGGACTTAGGGTCAGAAGGC |
| --- | --- |
| Universal 5′ primer | GCGAGCACAGAATTAATACGAC |
| β-actin-Forward | CTCCATCCTGGCCTCGCTGT |
| β-actin-Rerverse | GCTGTCACCTTCACCGTTCC |
| U6-Forward | CTCGCTTCGGCAGCACA |
| U6-Rerverse | AACGCTTCACGAATTTGCGT |
| pri-miR-422a-Forward | TGCATACCTCATTGGTGAGCAT |
| pri-miR-422a-Reverse | AGCCAAGCTAGGATAGCCA |
| pre-miR-422a-Forward | GAGAGAAGCACUGGACUUAG |
| pre-miR-422a-Reverse | CCAGGGAGGACAAAGCTTGGC |
| HK3-Forward | TGACAAGGGCACAGCTACAG |
| HK3-Rerverse | GAGTCACCCACAAAACACGC |
| PDP1-Forward | TCACTGCGCAGCCAATCG |
| PDP1-Reverse | GGCATGGCATCAGAGAACAG |
| PKM2-Forward | TCTGACCCCATCCTCTACCG |
| PKM2-Reverse | AGATCTTGCTGCCCACTTCC |
| PDK1-Forward | CCGCTCTCCATGAAGCAGTT |
| PDK1-Reverse | TGAACGGATGGTGTCCTGAG |
| PDK2-Forward | ATGGCAGTCCTCCTCTCTGA |
| PDK2-Reverse | CACCCACCCTCTTC CTAACA |
